# Upcycling Agricultural Waste for Functional Interfaces: Yellow Onion Skin-Derived Dyes for Cellulosic Materials

**DOI:** 10.1021/acsomega.5c05183

**Published:** 2025-08-11

**Authors:** Ritesh Sharma, Peppi Toukola, Juha Jordan, Julia Vuorinen, Ngoc Huynh, Nikita Durandin, Mikko Herrala, Anja Primetta, Jaana Rysä, Monika Österberg, Paula Nousiainen, Päivi Laaksonen, Riikka Räisänen

**Affiliations:** † School of Chemical Engineering, Department of Bioproducts and Biosystems, 174277 Aalto University, Vuorimiehentie 1, 02150 Espoo, Finland; ‡ Craft Science, 3835 University of Helsinki, Siltavuorenpenger 5, 00014 Helsinki, Finland; § HAMK Tech Research Unit, 3700 Häme University of Applied Sciences, Vankanlähde 9, 13100 Hämeenlinna, Finland; ∥ Faculty of Engineering and Natural Sciences, 7840 Tampere University, Korkeakoulunkatu 6, 33720 Tampere, Finland; ⊥ School of Pharmacy, 205537 University of Eastern Finland, Yliopistonrinne 3, 70211 Kuopio, Finland

## Abstract

The growing environmental and health concerns associated with synthetic dyes have increased interest in natural alternatives. This study investigates the multifunctional properties of yellow onion skin extract as a sustainable dye source for cellulosic substrates, assessing the extract’s functional properties beyond coloration. The extract was applied to premordanted cellulose nanofiber films (CNF) with a concentration of 2 g/L and knitted cotton fabric 0.125 g/L at 80 °C for 60 min, and its impact on UV protection, antioxidant properties, and dye stability was investigated. To enhance adsorption, biobased polyelectrolytes, including chitosan, tannic acid, and organic acids, were used as mordants and compared to metal mordants. Surface interactions between the cellulosic substrate and dye were studied using in situ monitoring to obtain data on the dyeing conditions for optimization. The dyed CNF films demonstrated excellent UV-shielding properties with up to 90% UV blocking and 82% DPPH radical scavenging activity, making them promising for protective packaging solutions. For cotton fabric, the dye concentration was low (0.125 g/L), resulting in relatively low color depth (K/S 1.33–5.59) with moderate (LF 3–5) fastness properties. Our study of KeratinoSens (OECD Test Guideline 442D equivalent) suggests a potential skin sensitizing effect of the yellow onion dye at higher concentrations. This needs further evaluation and studies. All together, these findings highlight the potential of yellow onion skin waste as a functional dye for applications in textiles and packaging that require enhanced surface properties.

## Graphical abstract

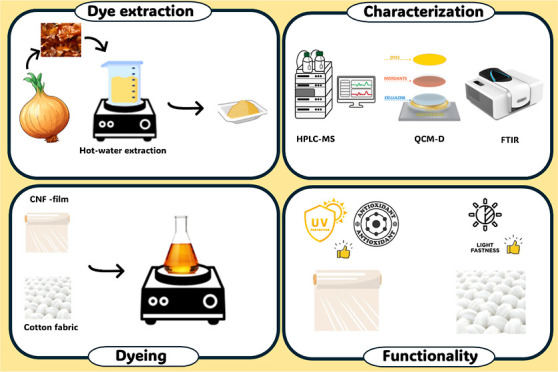

## Introduction

Natural dyes were once widely used in textiles, cosmetics, and various food products before being replaced by synthetic dyes.^1,2^ This transition was driven by the advantages of synthetic dyes over natural dyes, including lower costs, streamlined production methods, and superior overall performance.^3,4^ In recent years, there has been a renewed interest in identifying potential side streams and alternative sources for natural dye production, driven by the need for sustainable and environmentally friendly options to replace the oil-based derivatives prevalent in the dyeing industry.^5^ This interest is motivated by the increasing awareness of the health and environmental impacts associated with synthetic dyes, prompting a shift back to more sustainable and eco-friendly alternatives.^6,7^

Cellulosic fibers have inherently low affinity toward dyes because of their chemical structure and lack of binding sites, making it more challenging to achieve sufficient dye uptake.^8,9^ Therefore, to improve dye fixation metal mordants are commonly used.^10^ However, the common metal mordants that are used in the dyeing process come with significant environmental and health issues.^11,12^ To address this challenge, biopolymeric compounds including polysaccharides and polyphenols, are utilized in creating sustainable alternatives to conventional dyeing technologies for cellulosic fibers.^13,14^ In particular, biopolymers like chitosan and tannic acid, both of which can be derived from food processing industry waste, have shown great promise as biobased mordants because of their ability to enhance dye fixation and impart additional functionalities like UV-protection, antimicrobial, and antioxidant properties.^15–18^ Chitosan, which is extracted from crustacean shell waste,^19^ and tannic acid, abundantly found in agro-industrial byproducts like fruit peels and nut shells,^20^ not only can improve the dye uptake but it can also contribute to waste valorization. Recent advancements in natural dyeing have shown that biomordants such as chitosan, tannic acid, and organic acids provide an eco-friendly alternative to conventional metal mordants such as alum and iron sulfate in terms of dye uptake and binding strength.^21–23^ For instance, chitosan has been reported to act as both a mordant and a film-forming agent, enhancing color fixation and contributing antioxidant and antimicrobial properties to dyed substrates.^24^ They interact with natural dyes, facilitating better binding to cellulosic substrates, thereby enhancing the overall performance of the dyed materials.^23,25^

Natural dyes not only offer color but also additional functional characteristics, such as antimicrobial, antioxidant, deodorizing, and UV-protective properties, which enhance their multifunctionality and value as dyes.^26,27^ Various natural dyes have already shown potential applications in textiles, cosmetics, smart packaging, and dye-sensitized solar cells.^28–31^ Among these, plant-based flavonoid-rich dyes have gained significant attention due to their ability to bind to cellulosic fibers, offering an eco-friendly alternative for textile and packaging applications.^32,33^ Despite their advantages, natural dyes face certain limitations such as relatively weaker color fastness properties, and challenges in achieving reproducible shades compared to synthetic dyes.^34–36^ Therefore, to address these challenges substantial technological advancement is required, starting from the dye extraction process to the application techniques, and the utilization of biobased mordants to enhance the dye–substrate interactions. Furthermore, the use of synthetic biology and valorization of agricultural waste, such as onion skins, presents promising strategies for producing biobased dyeing solutions.^37,38^

Onion (*Allium cepa*) production is substantial worldwide, with approximately 93.2 million tonnes produced annually across 170 countries.^39^ China and India are the largest producers, contributing about 24 and 21 million tonnes, respectively.^40^ Along with the high volume of onion production, a significant amount of onion skin waste is generated each year from the food processing industry. While this waste is a major concern, it has also been identified as a valuable source of bioactive compounds.^41,42^ Onion skins, particularly those obtained from yellow onions, are rich in flavonoids with quercetin being one of the main compounds responsible for their strong dyeing properties.^43^ They have also been shown to impart UV protection and antioxidant properties when applied to textiles and packaging materials,^32,44^ making them an attractive resource for natural dye production.

In this study, we explored yellow onion skin waste as a natural dye source, extending its application beyond textiles to packaging. While previous studies have primarily focused on its use for dyeing cotton,^13,37,45^ here we dyed CNF films to extend their application to biobased packaging. We characterized the extract of yellow onion, *A. cepa*, using LC–MS/MS and LC-HRMS, showing quercetin and its glucosides as the main flavonoids, along with previously unreported compounds. Furthermore, to gain insight into dye-mordant interactions on cellulosic surface, Quartz Crystal Microbalance with Dissipation (QCM-D) was employed, and in situ adsorption kinetics were studied to determine the optimal conditions for dyeing cellulosic substrates. We further assessed the functional properties of the dyed materials, including UV-shielding, antioxidant activity, and potential skin sensitization. This research contributes new understanding of the binding mechanisms between natural dyes and biobased mordants such as chitosan and tannic acid, enhancing dye uptake and stability on both fabric and film substrates. By valorizing agricultural and food industry residues, including onion skins, our work supports the development of circular, sustainable materials solutions and provides a scientific foundation for optimizing natural dyeing processes in both functional textile and packaging applications.

## Experimental Section

### Materials

Yellow onions from the variety Settonia (the extract is called Settonia in this article) cultivated in Siilinjärvi, Finland, were obtained from Kesko (Helsinki, Finland). A batch of mixed yellow onion varieties (the extract is called here as YOD), the cultivar and origin of which were unknown, was obtained from Kesko.

The fabric used in dyeing was bleached 100% cotton knitted fabric (structure single, 185 g/m^2^) sourced from Orneule (Orivesi, Finland). The mordants used were potassium aluminum sulfate (Alum, KAl_2_(SO_4_)_2_·12H_2_O, ≥98%, J.T. Baker), iron­(II)­sulfate (FeSO_4_·12H_2_O, ≥99%, Sigma-Aldrich), tannic acid (≥99%, J.T. Baker), chitosan (75–85% deacetylated, medium molecular weight, Sigma-Aldrich), oxalic acid (≥99%, Sigma-Aldrich), and citric acid (≥99%, Sigma-Aldrich). Glauber salt (Na_2_SO_4_·10H_2_O, technical grade, A. Wennström) was used for fabric dyeing. A 12–15 μm pore size filter (VWR, Finland) was used for filtration during the YOD extraction process.

HPLC grade methanol (MeOH, ≥99.9%), and acetonitrile (MeCN, ≥99.9%) were from Honeywell Riedel–de Haën, and formic acid (99–100%) from VWR International. Quercetin-3-*O*-glucoside, quercetin dihydrate (Extrasynthese), and quercetin (anhydrous, ≥95%, Sigma-Aldrich) were used as standards. Type 1 ultrapure water (Milli-Q, Merck KGaA) was used for chromatographic measurements. 2,2-Diphenyl-1-picrylhydrazyl (DPPH >97%, Tokyo Chemical Industry) was used for the radical scavenging test. Butylated hydroxytoluene (BHT, >99%, Sigma-Aldrich) and Hostanox O 3 P (powder, bis­[3,3-bis­(4′-hydroxy-3′-*tert* butylphenyl)­butanoic acid]-glycol ester, Clariant Produkte GmbH) were used as referencing standards.

## Methods

### Dye Extraction and Drying Processes

The yellow onion dye (YOD) (Settonia) was prepared in a large batch. The amount of 4.6 kg of yellow onion skins (Settonia) was extracted in water with an LR 1:10. The extraction started at 40 °C, whereafter the temperature was raised to 80 °C within 20 min and kept at 80 °C for 2 h. The extract was then filtered through a 140-mesh filter with the solid content of the extract being 3.5 w %.^13^ To obtain a powder, the extract was spray-dried with an industrial scale spray dryer (GEA Spray dryer Niro *P*6̅.3) using the following heating parameters: inlet temperature 175 °C, outlet temperature 75 °C, feeding % increasing from 61 to 75, pressure difference 125 mm H_2_O. The dried powder was used for FTIR and skin sensitization tests. The yield was 21% solid content/dry yellow onion biomass.

For fabric dyeing purposes, a separate extraction was carried out for Settonia. Here, for each fabric sample (10 g), 2 g finely powdered onion skins were added to 150 mL of distilled water, raising the temperature from 20 to 80 °C 2 °C/min and heated at 80 °C for 30 min, followed by filtration of the extract through a 140-mesh filter. Then, an additional 50 mL of water was added to obtain a dye liquor in a volume of 200 mL and thus a fabric-to-liquor ratio (LR) of 1:20 for the dyeing step, which resulted in a dye solid content of 0.125 g/L.^13^ This preparation procedure and the resulting concentration were chosen to avoid leftover dye in the dye liquor after dyeing.

The YOD solution was prepared through aqueous extraction of 25 g of yellow onion skins with 1:30 LR. Dried onion skins were crushed and submerged in deionized water (pH 5.8), and the mixture was heated at 80 °C for 1 h. The skins were removed by filtration through a metallic mesh and twice using a 12–15 μm pore size filter paper. The dye liquor was stored covered from light at 4 °C between experiments. The solid content of the YOD solution was determined as 2 g/L and utilized for cellulose nanofilm (CNF) dyeing.

To conduct analytical studies, YOD was obtained in powder form by freeze-drying the liquor using a Labconco FreeZone 2.5 L system, operating at a pressure of 0.250 mbar and a temperature of −40 °C for 24 h.

### High-Performance Liquid Chromatography-Diode Array Detection-Tandem Mass Spectrometry

The Settonia sample for high-performance liquid chromatography-diode array detection-tandem mass spectrometry (HPLC-DAD-MS/MS) analysis was prepared using ultrasound-assisted extraction. 1.67 g of powdered onion skins were extracted in 20 mL of 10% MeCN: MeOH, 85:15 (v/v) and 90% aqueous HCOOH 8.5% (v/v) mixture.

The analysis was performed with HPLC (Agilent 1100, Agilent Technologies, Santa Clara, CA, USA) hyphenated with a DAD detector and quadrupole ion trap mass spectrometer (Bruker Esquire 3000 plus, Bruker, Billerica, MA, USA) equipped with an electrospray ionization (ESI) source. Positive and negative ionization modes were used. A Phenomenex Gemini C18 column (15 mm × 4.6 mm, 3 μm) was used for the chromatographic separations. The mobile phase A consisted of aqueous HCOOH 8.5% (v/v) and B of MeCN: MeOH, 85:15 (v/v). The chromatographic and spectrometric conditions are described in detail in Räisänen et al., 2023.^13^

### High-Performance Liquid Chromatography–High Resolution Mass Spectrometry

To identify dyes in YOD, an HPLC-QTOF-MS device equipped with an ESI interface (Agilent 1260) in negative and positive ion detection modes was used. The chromatographic separation was achieved using the method described in Grande et al. 2023.^22^ Data was acquired using MassHunter workstation software version 7.0 (Agilent Technologies) at a mass range (*m*/*z*) of 100–1500 and mass accuracy of ≤5 ppm using external calibration.

### Fourier Transform Infrared

ATR-FT-IR spectra of the YOD and Settonia powders were measured with an FT-IR spectrophotometer (PerkinElmer Spectrum Two) equipped with attenuated total reflectance (ATR) over 4000–500 cm^–1^ at a resolution of 4 cm^–1^. The spectra averages of 16 scans were reported.

### Zeta Potential of YOD Extract

To determine optimal pHs for the dyeing liquors, the electrophoretic mobility of YOD in different pH buffer solutions was measured with Zetasizer Nano ZS 90 Solutions (Malvern Instruments). The zeta-potential data were obtained from the electrophoretic mobility data by applying the Smoluchowski model. All the measurements were performed in triplicate, and results were reported as an average followed by respective standard deviations.

### Preparation of CNF Films

CNF films were prepared from 1.95 wt % fluidized never-dried hardwood pulp suspension. The suspension was prepared as described in Österberg et al., 2013.^46^

The CNF suspension was diluted to 0.8 wt % solid content with deionized water, magnetically stirred at 22 °C for 2 h, and centrifuged (Thinky Conditioning Mixer, ARE-250) for 2 min at 2000 rpm to obtain a homogeneous solution. Freestanding films were produced by filtering a 100 mL CNF suspension (0.8 wt %) through a Durapore membrane filter with 0.22 μm pore size at a pressure of 2.5 bar using an air press Sartorius AG, SM 162 76 (Caver laboratory press). The films were then dried in a hot press (Fred S. Carver Inc.) at 100 °C for 2 h under a pressure of 2000 kg/cm^2^. Before use, the films were conditioned at 23 °C and 50% relative humidity for 24 h.

### Mordanting and Dyeing of CNF Films with YOD

The CNF films were immersed initially in a 1% acetic acid solution for 30 min, followed by a 60 min immersion in the mordant solution at a fiber-to-liquor ratio of 1:50. The CNF film samples were premordanted with various mordants, including chitosan (5 g/L), alum (8 g/L), FeSO_4_ (3 g/L), oxalic acid (5 g/L), and citric acid (8 g/L). The mordanting step was conducted at a temperature of 50 °C for 60 min, followed by dyeing at 80 °C for 60 min. More detailed information regarding CNF dyeing parameters can be found in Table S1. The dyeing of CNF films with YOD was carried out in a Textex TD130 Infrared Lab Dyeing Machine (Testex Instrument, China). Both the mordanting and dyeing processes maintained a material-to-liquor ratio of 1:50. In addition, a control sample without mordant and dye was prepared under the same conditions for comparison purposes. Following the dyeing process, all the samples were thoroughly rinsed with distilled water.

### Mordanting and Dyeing Cotton Fabric with Settonia

Each cotton fabric sample (10 g) was premordanted in 100 mL distilled water, i.e., fabric-to-liquor mass ratio of 1:10. The mordanting agents used were alum (4 g/L, 8% owf (on weight of fabric)), FeSO_4_ (1.5 g/L, 3% owf), and tannic acid (2.5 g/L, 5% owf). Further, a reference sample without mordant was treated similarly in the premordanting step where the temperature was raised to 80 °C for 30 min. Mordating liquor was removed and dye liquor (200 mL) was added together with glauber salt which produced a concentration of 1 g/L. For dyeing, the LR 1:20 was thus obtained. Samples were dyed at 80 °C for 60 min, pH being acidic and ranging from 3.3 to 4.1 between samples. The original Hanau Lintiest machine (Hanau, Germany) was used for dyeing.

Dyed fabrics were tested for their color fastness according to the following standards: ISO 105-C06:2021 for domestic and commercial laundering using AATCC detergent (WOB), the DW multi-fiber test fabric, and the A1S washing method at 40 °C for 30 min, ISO 105-B02:2014 for light fastness, following Method 2 with exposure times of 0, 24, 72, and 168 h, and ISO 105-X12:2016 for rubbing fastness.

### Determination of Color Depth by CIELab and K/S of Cotton Fabrics

The color of the dyed samples was measured as CIE L*, a*, and b* and reflectance values (Smith, 1997) using a Konica Minolta (Tokyo, Japan) CM-2600d spectrophotometer (illuminant D65, CIE 10° observer) and recording SCI (Specular Component Included) values. This type of color evaluation measures the total appearance independent of the surface conditions. The K/S value was calculated by the Kubelka–Munk eq 1

1 $$\frac{K}{S}=\frac{{\left(1-R\right)}^{2}}{R}$$

where *K* is the absorption coefficient of the fabric to be tested, *S* is the scattering coefficient of the fabric, and *R* is the reflectance at the maximum absorption wavelengths. The wavelength of 420 nm was used for reflectance values.

### QCM-D Monitoring

The adsorption kinetics of YOD and Settonia onto CNF thin films were analyzed using Q-sense E4 Quartz Crystal Microbalance (Q-Sense, Sweden) with Dissipation Monitoring (QCM-D) at 22 °C. To simulate the dyeing conditions of cellulose substrates such as cotton or other cellulose fibers, thin films of CNF were prepared on 14 mm QCM-D quartz crystal resonators with gold electrodes (Q-sense, Biolin Scientific, Sweden). The CNF thin films were prepared through a spin-coating process where polyethylenimine (PEI, *M*_w_ 50,000–100,000, Sigma-Aldrich) was applied as an anchoring agent to CNF fibrils. A CNF suspension, containing only the finest fibrils, was spin-coated onto resonators at 4000 rpm for 1 min. The adsorption kinetics were conducted with Settonia and YOD extract under varying conditions. Settonia dye was tested at pH 4 using four different mordants: chitosan, tannic acid, FeSO_4_, and alum. In contrast, YOD was evaluated using only chitosan as a mordant at two pH levels, pH 4 and pH 5.8. The optimum pH of chitosan as a mordant was further evaluated for YOD at two pH values based on the variation in zeta-potential data. For both dyes, CNF films were first equilibrated with a 1% sodium acetate buffer until a stable baseline was achieved. Following this, various mordant solution at specified pH with a concentration of 0.1 g/L was injected into the chamber, and the resonance frequency (5 Hz) was monitored for 30 min. Once a plateau in the frequency shift was reached, which indicates full adsorption, the excess mordant was removed by rinsing the chamber with the same buffer solution. Subsequently, the dye solutions (0.1 g/L) were introduced under their respective conditions to evaluate adsorption kinetics on the mordant-coated sensors with monitoring continuing for 90 min. A final rinse step was performed to remove any loosely bound dye.

To quantify the sensed mass adsorbed onto a CNF film surface using QCM-D, the Sauerbrey equation was applied^47^

2 $$\Delta m=-C\times \frac{\Delta f}{n}$$

Where Δ*m* is the change in mass per unit area (ng/cm^2^), C is the sensitivity constant, 17.7 ng/cm^2^ for gold crystals, Δ*f* is the observed change in frequency (Hz) during the adsorption process, and *n* is the oscillation frequency overtone number.

### Measuring UV-Protecting Properties and UPF Values of Dyed CNF and Cotton Fabrics

The ultraviolet protection factor (UPF) values of the dyed CNF film samples and cotton fabrics were determined. In order to calculate the UPF for CNF films, the spectral transmittance was measured within the wavelength range spanning from 200–600 nm using an Agilent Cary 5000 UV–vis–NIR spectrophotometer. For cotton fabrics, the spectral absorbance in the 290–600 nm wavelength range was measured using a Shimadzu UV-3600 UV–vis–NIR spectrophotometer with an integrating sphere. From the measured absorbance values, spectral transmittance for the cotton fabrics was calculated using the following eq 3.

3 $$T_{\lambda }=10^{2-A_{\lambda }}$$

Where *T*_λ_ is the spectral transmittance and *A*_λ_ is the spectral absorbance at a single wavelength.

Following AS/NZS 4399:2017 guidelines, the UPF values of all dyed CNF samples and cotton fabrics were determined by measuring their total spectral transmittance within the 290–400 nm wavelength scale. UPF values ranging from 15 to 24 indicate good protection, values between 25 and 39 denote very good protection, while a UPF of 40 or higher signifies excellent protection.^48^ The UPF values below 15 are considered insufficient UPF protection.

The UPF, which represents the average effective ultraviolet radiation (UVR) irradiance of unprotected skin divided by that of dyed fabric-protected skin, was determined using the following eq 4.

4 $$UPF=\frac{\Sigma_{290}^{400}E_{\lambda }S_{\lambda }\Delta_{\lambda }}{\Sigma_{290}^{400}E_{\lambda }S_{\lambda }T_{\lambda }\Delta_{\lambda }}$$

Where, *E*_λ_ is the CIE erythemal spectral effectiveness, *S*_λ_ is the solar spectral irradiance, *T*_λ_ is the spectral transmittance of each sample, and Δ_λ_ is the wavelength step.

### Antioxidant Activity Testing

The radical scavenging activity of YOD and dyed CNF films was determined by using a spectroscopic 2,2-diphenyl-1-picrylhydrazyl (DPPH) assay, measuring the decrease in absorbance at 517 nm as the stable free radical in methanol was consumed. For the extract, a fresh stock solution of 0.1 mM DPPH radicals in methanol was prepared. Then, 2.950 mL DPPH radical solution absorbance was registered (*A*_1_) followed by the addition of 0.05 mL of the dye extract in methanol with various concentrations of 0.5, 1, 2, 4, and 6 mg/mL. The absorbance at 517 nm was measured at 1 min intervals using an Agilent Cary 5000 UV–vis–NIR spectrophotometer. Each analysis was conducted in triplicate. The results were expressed as the percentage of DPPH radicals scavenged within 30 min of reaction time. BHT and Hostanox were used as reference antioxidants.

Furthermore, the effectiveness of the dye extract was measured using the inhibitory concentration 50% method (IC_50_). A lower IC_50_ value indicates a more potent antioxidant, as less of the substance is needed to achieve 50% inhibition of the free radicals.^49^ The IC_50_ was calculated from the graph by plotting the scavenging percentage against the test sample concentration. This metric provides a clear and quantifiable measure of the antioxidant potency of the YOD extract.

For the CNF films, first, the sample specimen (5 × 20 mm) was immersed in 2 mL of 0.1 mM DPPH solution and left to react in a dark room at 22 °C. The samples were continuously rotated to ensure proper mixing throughout the reaction, and the reaction was carried out for 30 and 60 min. After the reaction time, the absorbance of the supernatant was measured at 517 nm. The radical scavenging activity (RSA) percentage was calculated using the following equation

5 $$RSA\;\left(\%\right)=\frac{A_{1}-A_{2}}{A_{1}}\times 100$$

Where *A*_1_ is the initial absorbance at the beginning of the reaction (absorbance of DPPH/methanol solution at 517 nm), and *A*_2_is the absorbance after 30 and 60 min of reaction time.

### Measuring Skin Sensitization Potential of *A. cepa*

A preliminary study with a commercial instaCELL KeratinoSens assay kit (acCELLerate GmbH, Germany) was performed to assess the skin sensitization potential of Settonia and YOD. The assay is based on luciferase Nrf2 gene activation in skin keratinocytes which is considered as the second key event in the skin sensitization Adverse Outcome Pathway (AOP, OECD 2022). The assay-ready keratinocytes were thawed and seeded onto 96-well plates with 10,000 cells per well and preincubated for 24 h in a humidified incubator (MCO-170M-PE, PHC Corporation, PHCbi, Japan), with 5% CO_2_, and a temperature of 37 °C, after which the cells were exposed for 48 h. For the experiments, 12.5 mg Settonia and 5.0 mg YOD powders were diluted into dimethyl sulfoxide (DMSO, Sigma-Aldrich) on a limit of solubility to prepare the following stock solutions: 12.5 mg/mL for Settonia and 5.0 mg/mL for YOD. Further, 12 concentrations from 0.06 to 125 μg/mL for Settonia and from 0.02 to 50 μg/mL for YOD were used for the exposures with three parallel wells per concentration. In addition, 1% DMSO was used as a vehicle control, and 15.63–250 μM ethylene glycol dimethacrylate (EGDMA, acCELLerate GmbH) was used as a positive control in the study.

After 48 h of exposure, cell viability was defined by adding 20 μL of resazurin to each well. After 4 h of incubation at 37 °C, fluorescence was measured with a microplate reader (Hidex Sense 425–301 microplate reader, Hidex, Finland). Immediately after the measurement, the exposure medium was removed, and the cells were washed once with 50 μL Dulbeccós phosphate buffer saline (DPBS, Gibco), after which 50 μL of DPBS and 50 μL of One-GloTM reagent were added per well. After 20 min incubation at 22 °C in the dark, luminescence was measured with an integration time of 1 s/well. The induction result is considered positive if gene activation is increased by 50%.

## Results and Discussion

### Chemical Characterization of the Extracted Yellow Onion Dye YOD and Settonia

The goal of HPLC-MS analysis was to thoroughly characterize the chemical composition of the YOD extracts. Understanding the detailed composition and properties of these compounds is crucial for optimizing their use as natural dyes. Accurate identification and characterization enable the development of more effective extraction and dyeing processes and improve the overall quality and performance of the dyed materials. The UV–vis chromatogram obtained from the HPLC-DAD-MS/MS analysis of the Settonia sample is presented in Figure 1, while the HPLC-MS TIC chromatogram for the YOD extract is provided in Figure S1. A total of 26 secondary metabolites were tentatively identified in the Settonia sample. A summarized list of annotated compounds, along with their structural information, retention time, UV–vis characteristics, and pseudomolecular ions acquired with MS/MS and QTOF-MS (from the YOD sample) is presented in Table 1. Compounds are numbered according to their elution order. More detailed MS/MS characteristics of the compounds can be found in Table S2. Tentative identifications of the compounds in the samples were done based on their UV–vis λ_max_ values, mass-to-charge ratios of the detected pseudomolecular ions, and their fragmentation patterns.

**Figure 1:**
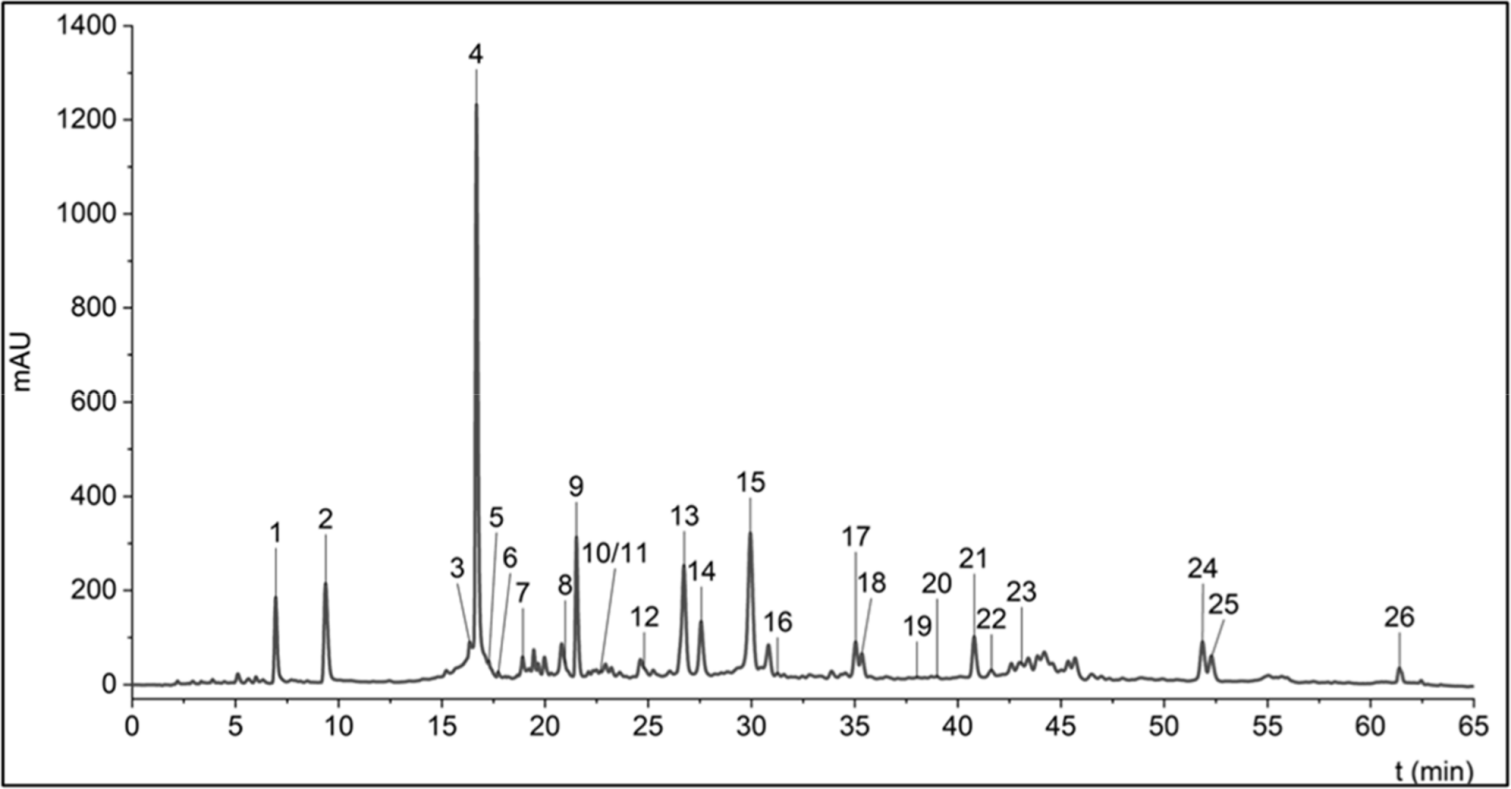
HPLC–UV–vis chromatograms of the Settonia sample monitored at 280 nm. The numbered peaks of the tentatively identified compounds are presented in Table 1.

**Table 1:** HPLC-DAD-MS/MS Data of the *A. cepa* (Settonia) Sample and the Tentatively Identified Compounds

no.	*t*_R_ (min)	λ_max_ (nm)	[M + H]^+^ (*m*/*z*)	[M – H]^−2^ (*m*/*z*)	HRMS (*m*/*z*)^c^	molecular formula	tentative identification	references
**1**	6.9	228, 260, 294	155	153	153.0225	C_7_H_6_O_4_	protocatechuic acid	^50–52^
**2**	9.4	230, 294	199	197	197.0091	C_8_H_6_O_6_	trihydroxy-phenylglyoxylic acid	^51–53^
**3**	16.3		627	625	625.1405	C_27_H_30_O_17_	quercetin-di-*O*-hexoside	^13,50,54,55^
**4**	16.7	234, 294, 320sh	319	317	317.0303	C_15_H_10_O_8_	2-(3,4-dihydroxybenzoyl)-2,4,6-trihydroxy-3(2H)-benzofuranone	^13,50,56^
**5**	17.3		627	625	625.1405	C_27_H_30_O_17_	quercetin-di-*O*-hexoside	^50,54,55^
**6**	17.7		641	639	639.1561	C_28_H_32_O_17_	isorhamnetin-di-*O*-hexoside	^50,54,55^
**7**	19.0	242, 290	599	597	597.1823	C_27_H_34_O_15_	phloretin-di-*C*-hexoside^b^	^57,58^
**8**	21.0		627	625			quercetin derivative	^54^
**9**	21.5	254, 265sh, 310sh, 366	465	463	463.0880	C_21_H_20_O_12_	quercetin-*O*-hexoside	^13,50,54,55^
**10**	22.7			615	615.0987	C_28_H_24_O_16_	quercetin galloyl hexoside^b^	^59^
**11**	22.7		479	477	477.1045	C_22_H_22_O_12_	isorhamnetin-*O*-hexoside	^50,55^
**12**	24.8		551			C_24_H_22_O_15_	quercetin malonyl hexoside^b^	^54,60^
**13**	26.7	240, 250sh, 292	455	453	453.0489	C_22_H_14_O_11_	protocatecoyl quercetin	^50,55^
**14**	27.6	240, 250sh, 292	455	453		C_22_H_14_O_11_	protocatecoyl quercetin	^50,56^
**15**	30.0	256, 298, 372	303	301	301.0351	C_15_H_10_O_7_	quercetin^a^	^13^
**16**	31.3		765	763	763.1174	C_36_H_28_O_19_	quercetin dimer hexoside	^50,56^
**17**	35.0	256, 273, 304, 370	765	763		C_36_H_28_O_19_	quercetin dimer hexoside	^13,50,56^
**18**	35.3	256, 274, 303, 366	765	763		C_36_H_28_O_19_	quercetin dimer hexoside	^13,50,56^
**19**	38.0		287	285	285.0428	C_15_H_10_O_6_	kaempferol	^50,54^
**20**	39.0		317	315	315.0303	C_16_H_12_O_7_	isorhamnetin	^50,54^
**21**	40.8	250, 272, 304, 364	765	763	763.1174	C_36_H_28_O_19_	quercetin dimer hexoside	^13,50,56^
**22**	41.6	250, 289sh, 300, 366	603	601			quercetin dimer or similar	^50^
**23**	43.1		765	763		C_36_H_28_O_19_	quercetin dimer hexoside	^50,56^
**24**	51.9	250, 272, 302, 364	603	601		C_30_H_18_O_14_	quercetin dimer	^50,56^
**25**	52.3	250, 272, 302, 364	603	601		C_30_H_18_O_14_	quercetin dimer	^50,56^
**26**	61.4	251, 275sh, 302, 360	903	901		C_45_H_26_O_21_	quercetin trimer	^50,56^

a Identification with a standard, sh = shoulder.

b Not previously seen in *A. cepa*.

c HRMS-ESI in negative ionization mode of YOD detected at <5 ppm mass accuracy.

Quercetin and its derivatives were found to be the major phenolic constituents in the yellow onion skins in question which is in accordance with our previous findings regarding the Settonia sample,^13^ as well as existing literature.^50,55,56^ However, when comparing these results to the red onion (*A. cepa*) extract, as previously reported by Grande et al.,^22^ there is a notable difference in several aspects. Red onion extract was characterized by significant anthocyanin content, particularly cyanidin derivatives contributing to the purple color. These compounds were absent in the yellow onion extract. Instead, the most prominent flavonoid compounds detected were quercetin (**15**) and quercetin-*O*-hexoside (**9**). Other quercetin derivatives included protocatecoyl quercetin (**13**, **14**), quercetin dimer (**24**, **25**), and quercetin dimer hexoside (**17**, **18**, **21**, **23**). Kaempferol (**19**) and isorhamnetin (**20**) were detected as minor flavonoids. The most abundant phenolic acid was protocatechuic acid (**1**), while peaks **2** and **4** were tentatively identified as two degradation products of quercetin: trihydroxy-phenylglyoxylic acid and 2-(3,4-dihydroxybenzoyl)-2,4,6-trihydroxy-3­(2*H*)-benzofuranone, respectively. Additionally, three compounds previously unreported in *A. cepa* were tentatively identified, i.e., phloretin-di-*C*-hexoside (**7**) ([M – H]^−^ at *m*/*z* 597.1823), quercetin galloyl hexoside (10) ([M – H]^−^ at *m*/*z* 615.0987), and quercetin malonyl hexoside (**12**) ([M + H] ^+^ at *m*/*z* 551). Compounds **7** and **10** were identified by their accurate masses detected in the analysis of the YOD sample and by the MS/MS data acquired in the analysis of the Settonia sample. Compound **12** was detected only in Settonia and was characterized according to its MS/MS data.

The MS/MS spectra generated by phloretin-di-*C*-hexoside (**7**) exhibited typical fragmentation behavior of *C*-glycosylated compounds and were assigned according to the literature.^57,58^ Quercetin galloyl hexoside (**10**) showed neutral losses of 152 Da (galloyl moiety) and 162 Da (hexoside moiety), producing a fragment ion at *m*/*z* 301 (quercetin). Compound **12** exhibited parent ions [M + H]^+^ at *m*/*z* 551 and [M – H – CO_2_]^−^ at *m*/*z* 505 with a cleavage of 248 Da (malonyl hexoside) in the positive ionization mode, leading to the tentative identification of quercetin malonyl hexoside in agreement with the literature.^60^

The most prominent flavonoid compounds were identified in YOD based on their molecular mass HRMS analysis (Table 1). However, unidentified peaks also remained showing that the onionskin extract compositions were not identical between different sources.

### FT-IR Spectroscopy

The FT-IR spectroscopic analysis presented in Figure 2 provided information on the variation between isolated fractions spectra for both the freeze-dried YOD powder and the Settonia powder.

**Figure 2:**
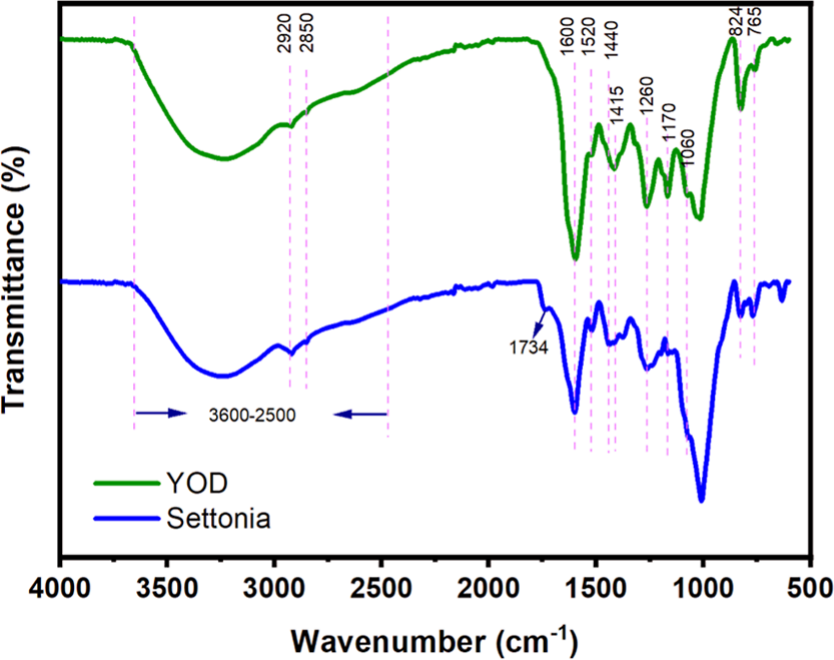
FT-IR spectra of YOD and Settonia.

The analysis serves as fingerprint data to evaluate the success of the pretreatments and to identify functional groups in the complex mixtures. In both YOD and Settonia, the broad band at 2500–3600 cm^–1^ corresponds to the O–H stretching vibration characteristic for hydroxyl groups and intermolecular hydrogen bonds.^61,62^ The bands around 2920 and 2850 cm^–1^ are associated with C–H stretching vibrations, typically originating from aliphatic hydrocarbon groups suggesting the presence of −CH, –CH_2,_ and –CH_3_ groups within the molecular structure of the compounds present in the mixtures.^63,64^ Additionally, both Settonia and YOD show broad absorption bands at 1618, 1600, and 1520 cm^–1^ that could be attributed to conjugated carbonyl group C=O together with the aromatic ring C=C skeleton vibrations typical for flavanols.^65^ The presence of these common bands in both samples suggests similar fundamental chemical structures, typical of aromatic ring skeletons of the polyphenolic compounds present in onion skins. Significantly, in YOD these bands were most prominent in the spectrum, indicating higher amounts of aromatic structures in the sample compared to Settonia.

In the fingerprint region, the C–H and C–O stretching and bending absorption bands were present, such as the aromatic ring C–H in-plane bending vibrations at 1440, and out-of-plane at 765, and 824 cm^–1^.^66^ The stretching vibrations of C–O are typically found between 1000– 1200 cm^–1^, and the phenol C–O hydroxyls were detected at 1170 and 1260 cm^–1^.^64^ However, Settonia shows an additional band at 1734 cm^–1^, which is typical for nonconjugated ester acids, possibly originating from pectin acetyl groups,^67^ and the highest signal in the spectrum was 1025 cm^–1^ characteristic of glycoside C–O stretching bands, suggesting a higher carbohydrate content in the sample in contrast to YOD.

These differences may result from the origin of the material and its extraction and drying procedures. However, more detailed studies would be needed to confirm the grounds for these differences. Understanding the variations is important for optimizing the extraction conditions and drying process to maintain the effectiveness of the dyeing batches and assist in their application as sustainable dyes. The higher carbohydrate content of the sample would lead to a higher amount of dye required for the dyeing process, as found in dyeing cotton fabric with Settonia extract.

### Effect of Mordant on In Situ Adsorption of YOD on CNF

In situ, adsorption measurements were performed using QCM-D to get insight into the adsorption mechanism of the flavonoid-based biodyes and mordants. CNF thin films were used to mimic both cotton fabric and cellulosic packaging materials. Further, to understand the role of charge density and distribution in the binding of YODs and cellulosic fibers, zeta-potential measurements for YOD and chitosan were conducted at different pH levels (Figure 3a).

**Figure 3:**
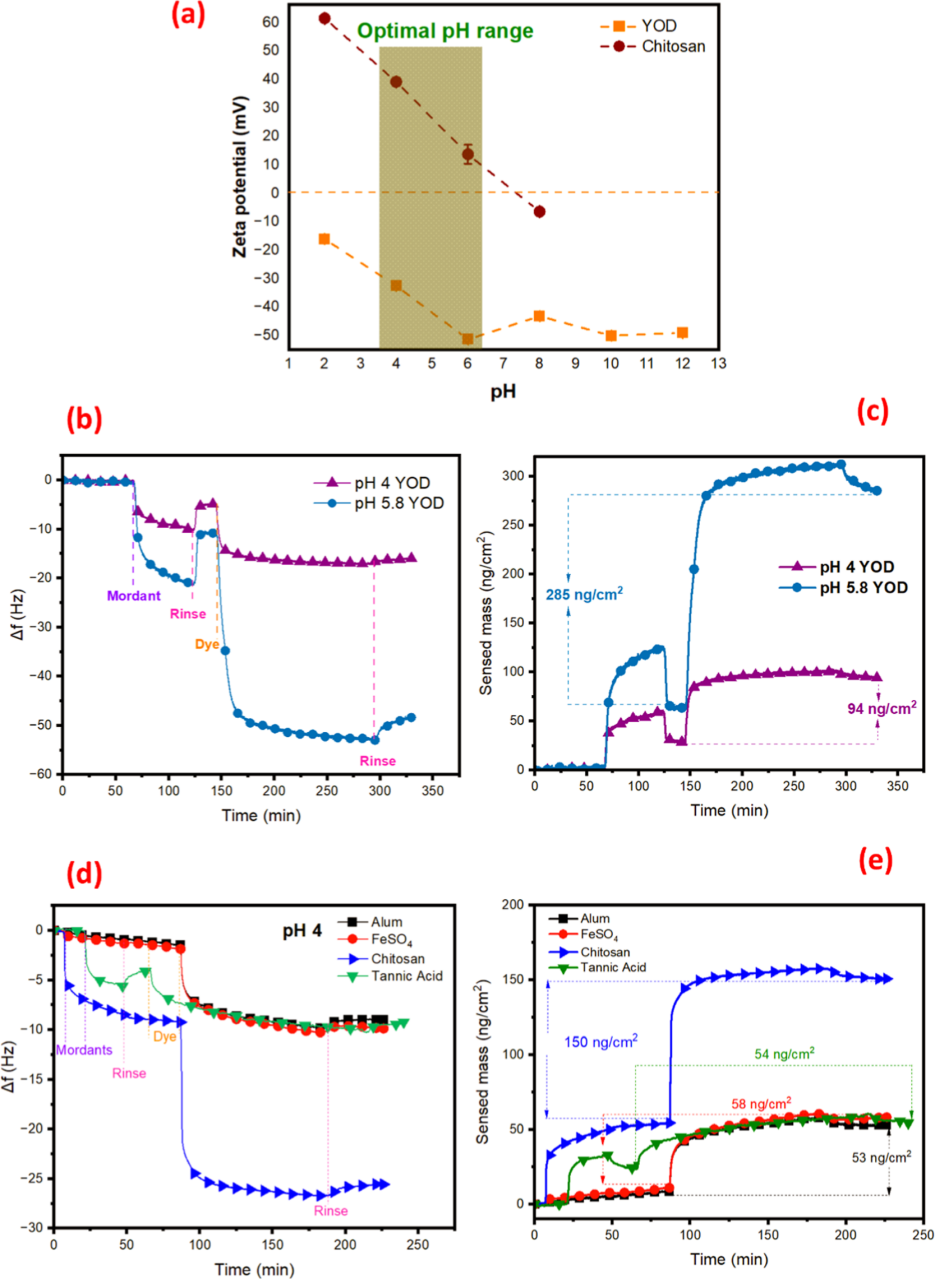
(a) Zeta-potential of YOD and chitosan as a function of pH. (b) Variation in oscillation frequency (third overtone) over time for YOD, obtained from QCM-D measurements, on CNF thin films using chitosan as a mordant at pH 4 and 5.8. (c) Mass adsorbed after rinsing as a function of time for YOD at pH 4 and 5.8, calculated using Sauerbrey equation (d) variation in oscillation frequency over time for Settonia, obtained from QCM-D measurements, on CNF thin films using chitosan, tannic acid, FeSO_4_, and alum as mordants at pH 4 (e) mass adsorbed after rinsing as a function of time for Settonia at pH 4, calculated using the Sauerbrey equation.

Zeta-potential measurements of YOD and chitosan at various pH levels offer important information about their charge behavior and stability. The measurements revealed that the chitosan solution exhibited positive zeta-potential values at acidic pH. However, with an increase of pH, a zero net charge was obtained leading to the precipitation of chitosan. This behavior aligns with the known chemistry of chitosan, which is more positively charged in an acidic environment due to the protonation of its amine groups.^22^ The YOD carried a negative charge in all the measured pH ranges of 2–12 with the lowest zeta-potential value observed between pH 5–6. The interaction between YOD and chitosan can be comprehensively explained through their respective zeta-potential behaviors. Particularly, between pH 5–6, the zeta potential measurements indicate a strong attraction expected between the negatively charged YOD and the positively charged chitosan, as the opposite charges facilitate binding. To understand more about the adsorption kinetics of chitosan with YOD and Settonia, QCM-D measurements were conducted for YOD at pH 4 and pH 5.8, and for Settonia at pH 4 also by using FeSO_4_ and alum as reference mordants. These pH levels were selected to observe how changes in charge affect the affinities and adsorption process. Compared to the previous study on the red onion extract, which contains both flavonoids and anthocyanins,^22^ the YOD exhibited a wider pH range. As reported by Grande et al., anthocyanins in red onion dye gradually hydrolyze at pH levels above 4, limiting their effective dyeing conditions to a broader pH environment. In contrast, YOD, which contains only flavonoids (Table 1), allowed a successful dyeing even at higher pH levels by maintaining better color stability and functionality.

The QCM-D data shown in Figures 3 and S2 illustrate the real-time adsorption behavior of both mordants and dyes on mordant-coated CNF thin films at different pH levels. The frequency shift provides insights into the adsorbed mass on the surface of the sensor, where negative frequency shifts indicate higher mass deposition. On the other hand, dissipation reflects the viscoelastic properties of the adsorbed layer. Higher dissipation indicates a softer, more viscoelastic layer, while lower dissipation suggests a more rigid and compact layer.

On adsorption of chitosan and YOD on CNF at pH 5.8, the most substantial frequency shifts were observed, indicating significant adsorption of both chitosan and YOD. Upon injection of chitosan, the frequency curve showed a downward trend, indicating a strong binding of positively charged chitosan onto the negatively charged CNF surface. At the same time, an increase in dissipation factor and steeper slope of the Δ*D*-versus-Δ*f* curve was also observed (Figure S2g), indicating more adsorption as well as the formation of a soft viscoelastic layer. This is consistent with the expected swollen, gel-like conformation of chitosan under mildly acidic conditions. After the rinsing step, the frequency and dissipation remained relatively stable, indicating that the adsorbed layer of chitosan adhered strongly to the surface with minimal desorption. Subsequently, when the dye was introduced, a second drop in frequency occurred, along with a rise in dissipation (Figure S2b), indicating the adsorption of YOD onto the predeposited chitosan layer. After the postrinsing step, only a slight rise in frequency was observed suggesting that most of the YOD remains bound, showing stable dye retention. The adsorption behavior of chitosan at pH 5.8 is likely due to electrostatic interactions between the positively charged chitosan and the negatively charged YOD, as shown by zeta-potential measurements. Additionally, the primary driving forces for the strong adsorption are likely entropic forces due to the release of counterions and water molecules surrounding the slightly hydrophobic phenolic compounds.^68,69^

In contrast, at pH 4, the adsorption of chitosan was moderate due to the stronger affinity toward CNF leading to the formation of an extended conformation in solution that results in a flat adsorbed layer. Consequently, smaller absolute values of frequency (Figure 3d) and dissipation shifts (Figure S2a) were observed. Although dye adsorption did occur at this pH, the total frequency shift was less pronounced, indicating reduced dye uptake compared to pH 5.8. While the chitosan is exceedingly positively charged at pH 4, the dye’s charge is less negative, resulting in diminished electrostatic interaction and hence reduced adsorption efficiency.

For Settonia, the adsorption kinetics were monitored using chitosan, tannic acid, FeSO_4_, and alum as mordants at pH 4. The choice of pH 4 for Settonia was driven by the need to evaluate different mordant’s efficacy under acidic conditions, which are effective in the dyeing processes of cotton.^13,22^ The QCM-D data indicated that the adsorption behavior varied significantly depending on the mordant used. Upon injection of the mordants, a noticeable decrease in frequency and increase in dissipation (Figure S2e) occurred in the case of chitosan, indicating the successful binding and formation of a robust stable layer. In contrast, the total frequency shift for the metal mordants like alum and FeSO_4_ (Figure S2c,d) was lower compared to that of chitosan. This is because polymers like chitosan and small molecules such as metal salts (alum and FeSO_4_) have different adsorption behaviors.^22,70^ Polymers, due to their hydrophilic nature, can bind water leading to notable shifts in frequency and dissipation.^71,72^ In contrast, metal ions like Fe^2+^ or Al^3+^ form a compact layer without water retention. This results in minimal dissipation and frequency shifts. Particularly, with quercetin, Fe^3+^ or Al^3+^ salts can form coordinated complexes via the 3′,4′-dihydroxy catechol group.^73^ This mechanism is common among known metal-flavonoid chelation mechanisms, which result in compact and less hydrated adsorption layers.^74^

After rinsing with a buffer solution to remove unbound mordants, Settonia dye was introduced. The subsequent frequency shifts observed from Figure 3d suggest that the Settonia dye adsorbed onto the chitosan-mordanted CNF films more effectively in comparison to tannic acid, FeSO_4,_ and alum-mordanted CNF films. In addition, the sensed mass curves were obtained using the Sauerbrey equation^47^ to compare the results of employing different pH systems. The sensed adsorbed mass values of YOD while chitosan was used as a mordant at pH 4, and pH 5.8, after the rinsing step, were estimated to be 94 and 285 ng/cm^2^, respectively. On the other hand, for Settonia with chitosan, FeSO_4_, tannic acid, and alum as mordants at pH 4, the estimated sensed adsorbed mass values were 150, 58, 54, and 53 ng/cm^2^, respectively.

These findings show that employing chitosan as a mordant at pH 5.8 led to a higher adsorbed amount of YOD compared to lower pH, i.e., pH 4, or using tannic acid, alum, and FeSO_4_ as mordants. The strong adsorption of YOD at pH 5.8 also aligns with the findings that chitosan exhibits optimal charge and interaction with dyes in mildly acidic conditions, promoting efficient dye-mordant binding through electrostatic interactions, as supported by the zeta-potential measurements and corroborated by previous studies.^75^ In summary, these results underscore the importance of selecting appropriate pH levels and mordants to optimize dye adsorption processes.

### Dyeing of Cotton Fabric Using Settonia as Dye

Chitosan is known to be an efficient mordant for cellulosic substrates because of its strong electrostatic interactions.^18,24^ However, it was not selected as a mordant for fabric samples in this study as preliminary experiments revealed a reduction in fabric flexibility due to stiffening.^13,22^ Instead, tannic acid was chosen as it has shown efficiency as a natural mordant in several applications with natural dyes.^16,76^

The results from Settonia dyed cotton fabrics using alum (S2-Al), iron sulfate (S3-Fe), and tannic acid (S4-Tann) as the mordants are presented in Table S3 in the Supporting Information. Sample S1-0 has no mordant and indicates the direct dye behavior of Settonia dye. The CIE L*, a*, and b* values indicate that iron sulfate as a mordant produced the darkest and deepest color, as evidenced by the lowest L* and highest K/S values (Table S3). The color obtained using natural mordant tannic acid resulted in a pale beige, similar to the color obtained when Settonia was applied as a direct dye. The low color strength of these samples, indicated by K/S values of 1.33, the lowest among the studied samples, highlights the challenge presented by tannic acid as a natural mordant for not improving the color. This aligns with the findings from Shibly et al.,^77^ who reported that tannic acid, due to its structural resemblance to flavonoid dyes, does not significantly enhance the color. Furthermore, tannic acid fails to add electrons in the color chromophore, unlike metal mordants. Therefore, the color remains pale with low K/S values. In contrast, alum, which contains Al^3+^ ions, effectively forms a complex with the flavonoid chromophore, adding electrons to the system and thus increasing the intensity of the obtained color, making it a preferable choice for applications requiring bright color. Also, as a positive ion, Al^3+^ is adsorbed by the negatively charged cotton fiber surface, attached onto it, and further draws negatively charged dye molecules onto the fiber surface, thus enhancing dye uptake. Similar results were obtained by Bhattacharya and Shah,^78^ who reported that the use of alum as a mordant can improve the dye uptake and enhance the depth of dyeing. On the other hand, iron sulfate, turned the color strong dark green with a K/S value of 5.59. The change in color is the result of iron–flavonoid complex formation, which shifts the color of the dye toward darker shades.^13,79^

The light fastness result showed that tannic acid-mordanted and direct dye samples exhibited the highest light fastness values (5, on a scale of 1–8). This can be attributed to the numerous hydroxyl bonds formed between the dye and the cellulose fiber in acidic conditions, which stabilize the dye structure and hence improve the resistance to photofading.^13,80^ Additionally, Phan et al.^81^ reported that the gallic acid moieties within tannic acid, known for their antioxidant properties, can act as a copigment by further strengthening their ability to withstand photodegradation. Similar observations regarding the use of tannic acid as a mordant for cotton dyeing were also reported by Bechtold et al.^82^ Furthermore, studies have shown that biomordants, such as tannins, enhance color stability by forming hydrogen bonds and π–π interactions (Figure 4) with dye molecules.^81^ In comparison, the lightfastness of alum-mordanted cotton was lower with a value of 3, while iron-mordanted cotton had a value of 4. The stronger metal complex formation between iron and flavonoid dyes likely contributes to its higher light fastness compared to alum.^76,83,84^ Metal mordants like iron form coordination bonds with flavonoid structures, reducing their susceptibility against photodegradation by stabilizing the chromophore system.^84,85^

**Figure 4:**
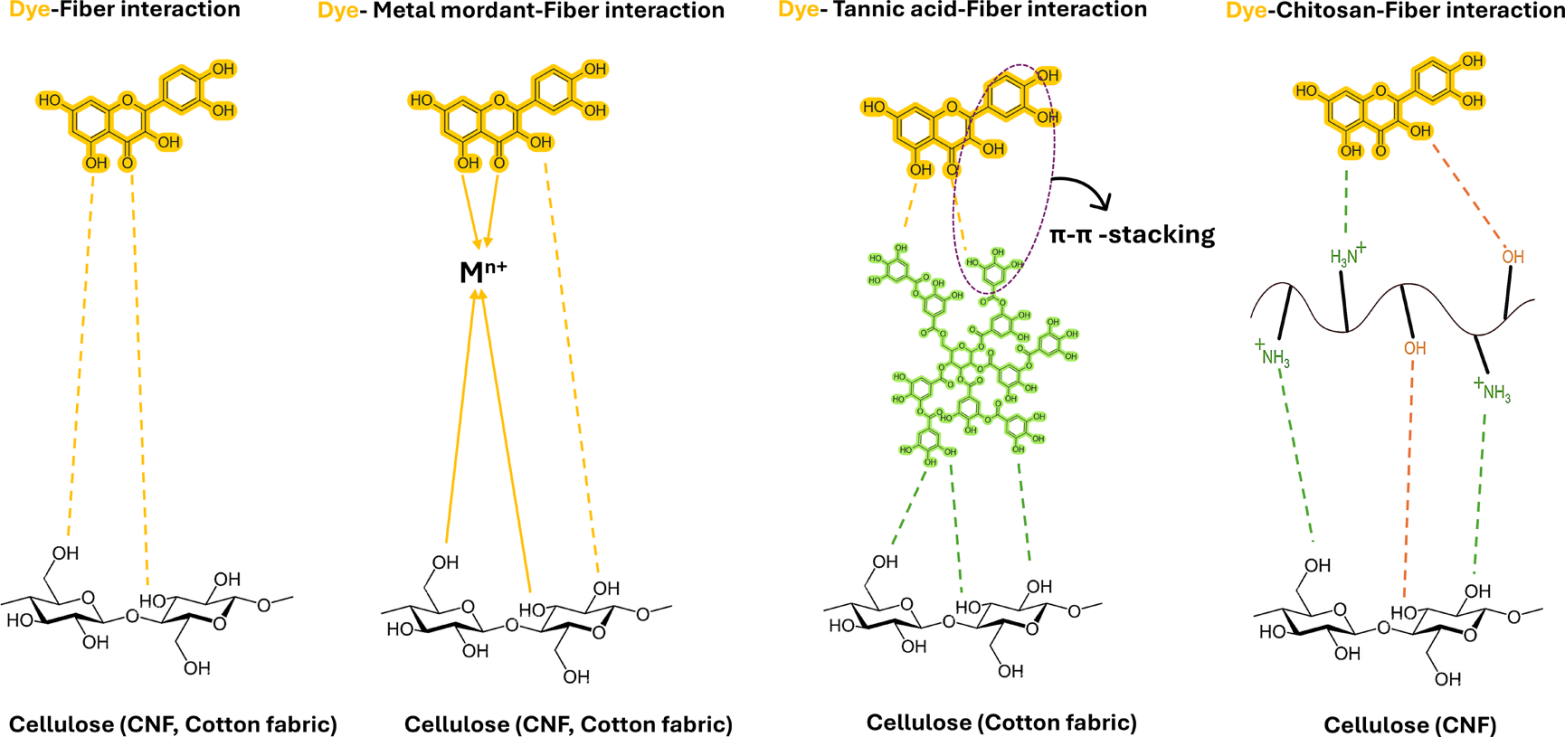
Schematic illustration of the interaction between YOD with quercetin as a model flavonoid compound, mordants (metal mordants M, chitosan, and tannic acid), and cellulosic fibers. The dashed lines indicate hydrogen bonding, arrows coordinate bonding with metals, and the circle aromatic π–π-stacking.

Washing fastness tests were performed at pH 10 and at pH 7. The dyeing was performed in an acidic environment, but the washing fastness test was performed in highly basic soap liquor following the established standard ISO 105-C06. Consequently, a clear color change was observed after washing, with the color change values being low with pH 10 detergent (2, 2/3, on a scale of 1–5, respectively (Δ*E* values high 26.60, 15.46, Table S3)). This color change was due to bathochromic shifts in the absorption spectra of the ionized dyes at alkaline pH.^86^ However, when washed using a neutral detergent (pH 7), less color change was observed in the dyed samples, with ratings improving to 3/4 and 4/5 (Δ*E* 10.11 and 3.85, respectively, Table S3). The Δ*E* value 2 is usually characterized as an observation limit to the human vision, meaning that a very slight color change was observed with the best color change values of YOD. In addition to color change, staining values were assessed and ranged from 4 to 5 (Table S3), suggesting a minimal dye transfer onto adjacent fabrics. These results show that for natural dyed textiles, milder (neutral) washing conditions should be applied to minimize color change and preserve dye integrity. Given the growing interest in natural dyes, revising the current textile care standard to include milder washing conditions would better align with current environmental and sustainability goals.

### Skin Sensitization

The potential skin sensitization effects of Settonia and YOD were assessed using the KeratinoSens assay. Settonia had no significant effect on cell viability (results not shown), nor reporter gene induction as presented in Figure 5.

**Figure 5:**
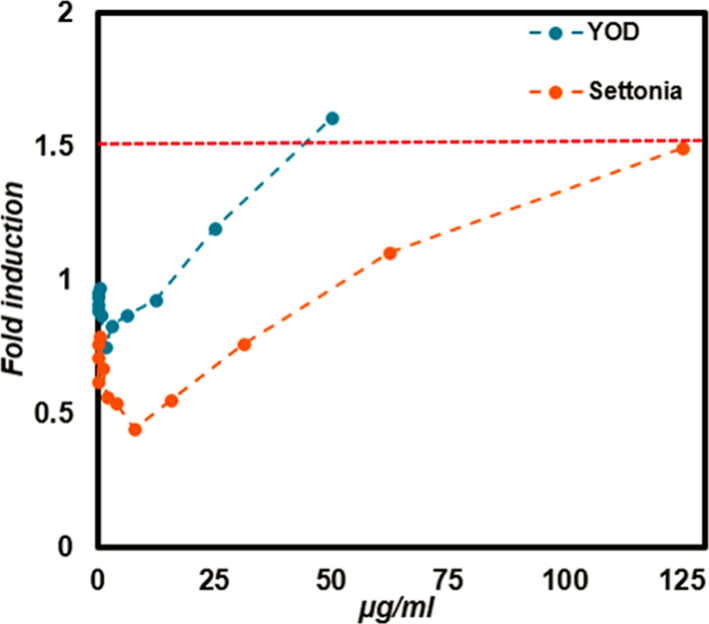
Skin sensitization assay shows the reporter gene induction compared to the control after 48 h exposure to YOD and Settonia, with a 1.5-fold increase marking the induction limit (red dashed line).

Moreover, there was an increasing trend in reporter gene induction with higher doses of Settonia, which may have resulted from precipitation at these concentrations. However, none of the doses exceeded the 50% induction limit. Instead, YOD caused 1.6-fold reporter gene induction compared to control at the highest concentration tested (50 μg/mL), without affecting cell viability, placing it within the borderline sensitization range (1.35–1.67-fold) as defined by Kolle et al. (2021).^87^ This result suggests that YOD may act as a potential skin sensitizer at higher concentrations, despite the presence of quercetin and related flavonoids that are generally considered dermatologically safe.^88,89^

Given the complex composition of yellow onion extracts, there is a possibility of mild sensitization under specific conditions. Therefore, while YOD shows potential as a natural textile dye, particularly due to its biobased origin, its usage in direct and continuous skin contact, such as clothing, should be approached with care. Furthermore, formulation control to restrict extract concentration in the final fabric, pretreatment to eliminate unbound compounds, and postdye washing techniques may all help to reduce sensitization concerns. However, there is a need to conduct additional studies utilizing other available assays for toxicity assessment and to fully understand the dermal safety of YOD extract.

### UV Shielding in Dyed CNF Films and Cotton Fabric

The UV–vis light transmittance spectra of the dyed CNF films were collected in the region of 200–600 nm (Figure 6a). The CNF film (Figure 6b) without a dye displayed the highest transmittance in the UV–vis region, indicating low UV-blocking ability. In contrast, the YOD-dyed CNF film mordanted with chitosan offers the highest level of UV protection with the lowest observed transmittance. Other tested mordants, such as FeSO_4_, alum, and organic acids also improved UV protection, although not as significantly as chitosan.

**Figure 6:**
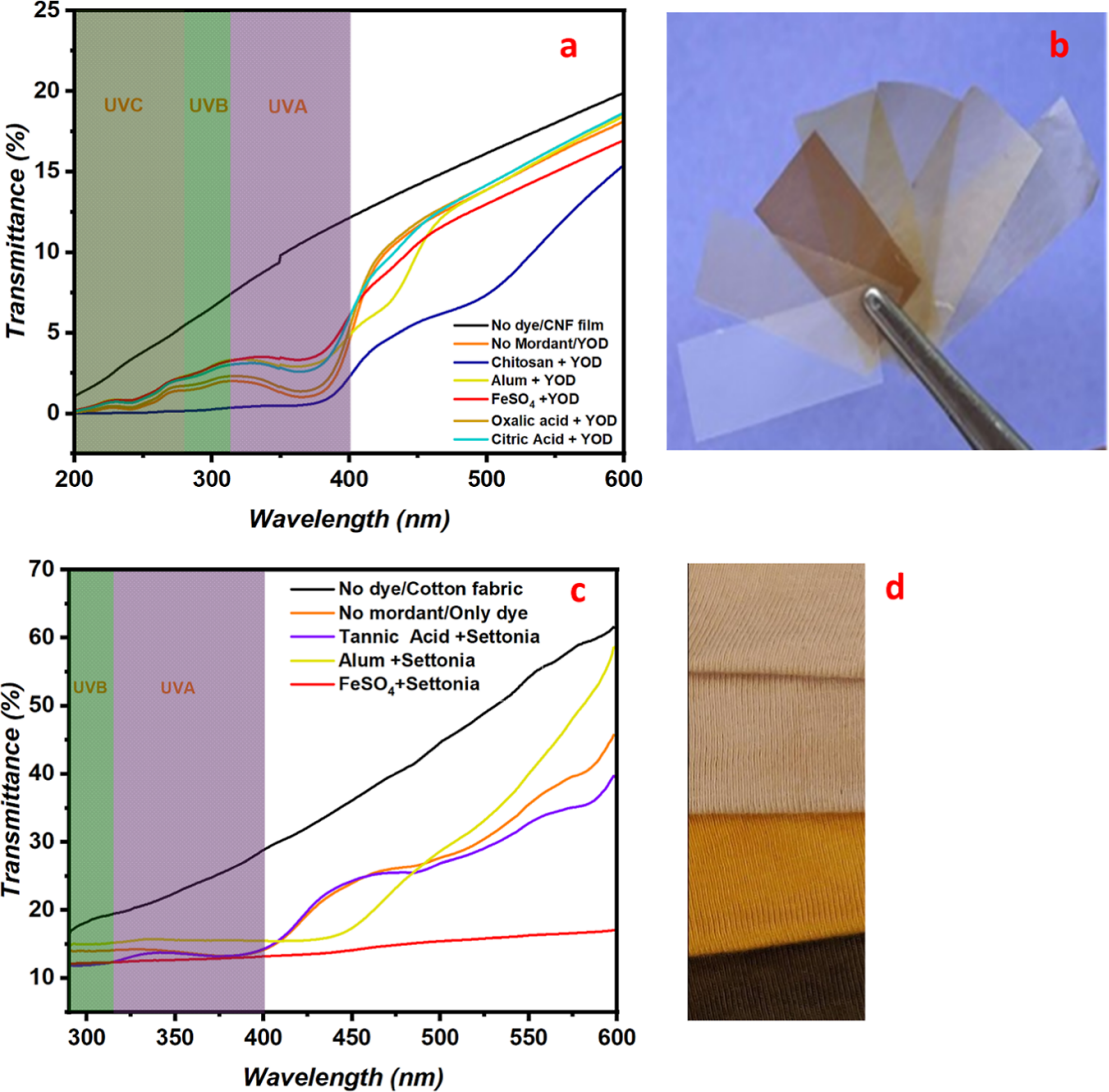
(a) UV–vis transmittance spectra of YOD dyed CNF films using chitosan, alum, iron sulfate, oxalic acid, and citric acid as mordants, (b) picture of YOD dyed CNF films using various mordants (films from left to right are in the same order as listed in the UV-graph), (c) UV–vis transmittance spectra of Settonia dyed cotton fabrics, (d) picture of Settonia dyed cotton fabrics (from top to bottom in the same order as listed in the UV-graph, nondyed fabric sample is not included).

The UPF values of dyed CNF films further validated these findings. The UPF values obtained for the CNF films dyed with YOD using various mordants range from very good to excellent (Table S3). The undyed CNF film exhibited a baseline UPF value of 11, indicating minimal UV protection. In comparison, the CNF film dyed with YOD alone (without any mordant) showed a substantial improvement, reaching a UPF of 63. Notably, the highest UV protection was achieved when chitosan was used as a mordant, with the UPF value increasing to 223, representing a ∼20-fold improvement over the undyed film and over 3.5 times higher than the film dyed without any mordant (Table S3). This finding highlighted the importance of YOD as a standalone UV-protective agent that can eliminate the need for mordants and hence simplify the CNF film dyeing process. Since packaging materials prioritize UV-shielding over wash fastness, such films offer a sustainable and practical solution for applications where durability against washing is not required.^90–92^

The results highlight chitosan as the most effective mordant, considerably enhancing the UV protection of CNF films dyed with YOD, in both the UV-A (at 315–400 nm) and UV–B (at 280–315 nm) regions. This observation can be attributed to the high affinity of chitosan as a positively charged polyelectrolyte to bind cellulose and further the flavonoids, as shown by the in situ absorption experiments (Figure 3). Chitosan’s capacity to bind YOD at pH 4 was double that of other mordants, and at pH 5.8 five times higher. The natural UV-absorbing properties of flavonoids, such as quercetin and its derivatives that are present in YOD, are known as the plant’s primary defense mechanism against solar radiation.^32,93^ This absorption capability originates from their aromatic backbone structures with double bonds conjugated to carbonyl chromophores.^32,91,94^ These compounds form a uniform layer on the film, effectively blocking UV radiation.^95^ In addition, the high surface area and strong hydrogen bonding ability of CNF films enhance dye adsorption, leading to effective UV shielding in the films.^46,96^ Furthermore, the CNF films have a highly entangled and dense network because of their nanoscale fibrillar structure, which results in reduced porosity and limited light penetration.^97,98^ These structural advantages combined with the UV-absorbing properties of flavonoids contribute to the superior UV-shielding performance of CNF films.

For the cotton fabric UV–vis light transmittance spectra were collected in the region of 290–600 nm (Figure 6c). The undyed cotton showed the highest transmittance in the UV–vis region by a substantial margin, indicating very minimal UV protection. In contrast, fabrics treated with mordants (alum, FeSO_4_, and tannic acid) showed reduced transmittance in the UV region, suggesting improved UV shielding. Among the mordants used, FeSO_4_-treated samples showed the lowest transmittance, indicating the highest UV-blocking performance, while alum and tannic acid treatments provided moderate protection.

The UPF values (Table S3) obtained for dyed cotton fabrics (Figure 6d) showed that FeSO_4_ and tannic acid mordanted samples achieved higher UPF values than the other samples, showing improved UV protection. Moreover, all dyed fabrics exhibited better UPF values than undyed cotton. However, despite improving the UV protective ability of cotton fabric, none of the dye-mordant combinations gave the fabric a satisfactory UV protection classification. This is likely due to the low dye concentration used in our study and insufficient dye penetration and weaker dye–fiber interactions, which are inherent challenges in the surface chemistry of cotton fabric. Additionally, knitted cotton fabrics have a porous structure that can also allow some UV rays to pass through the holes between the yarns and fibers reducing their UV-shielding ability.^99^ In addition, Wong et al. (2013) highlighted that the UV protection ability of a fabric relies not only on the presence of UV-absorbing agents but also on the choice of fabric parameters such as thickness, weight, and stitch density.^100^ Thicker and denser fabrics tend to offer better UV protection as they minimize light penetration.^101^ These factors highlighted the importance of optimizing both the dyeing processes as well as the fabric manufacturing process to provide an effective UV-shielding property to the cotton fabrics.

To overcome these limitations, advanced wet-processing technologies such as ultrasonic-assisted or microwave-assisted dyeing have shown potential in enhancing dye diffusion and fiber–dye interactions.^102^ Furthermore, plasma surface modification of cotton has been shown to increase surface energy and wettability, promoting better dye adsorption and uniform distribution across the fiber surface.^103^

### Antioxidant Activity

The result obtained from DPPH radical scavenging activity showed that the YOD extract exhibited an IC_50_ value of 3.96 ± 0.17 mg/mL, while BHT and Hostanox demonstrated IC_50_ values of 2.04 ± 0.11 mg/mL and 18.19 ± 1.01 mg/mL, respectively as presented in Figure 7a.

**Figure 7:**
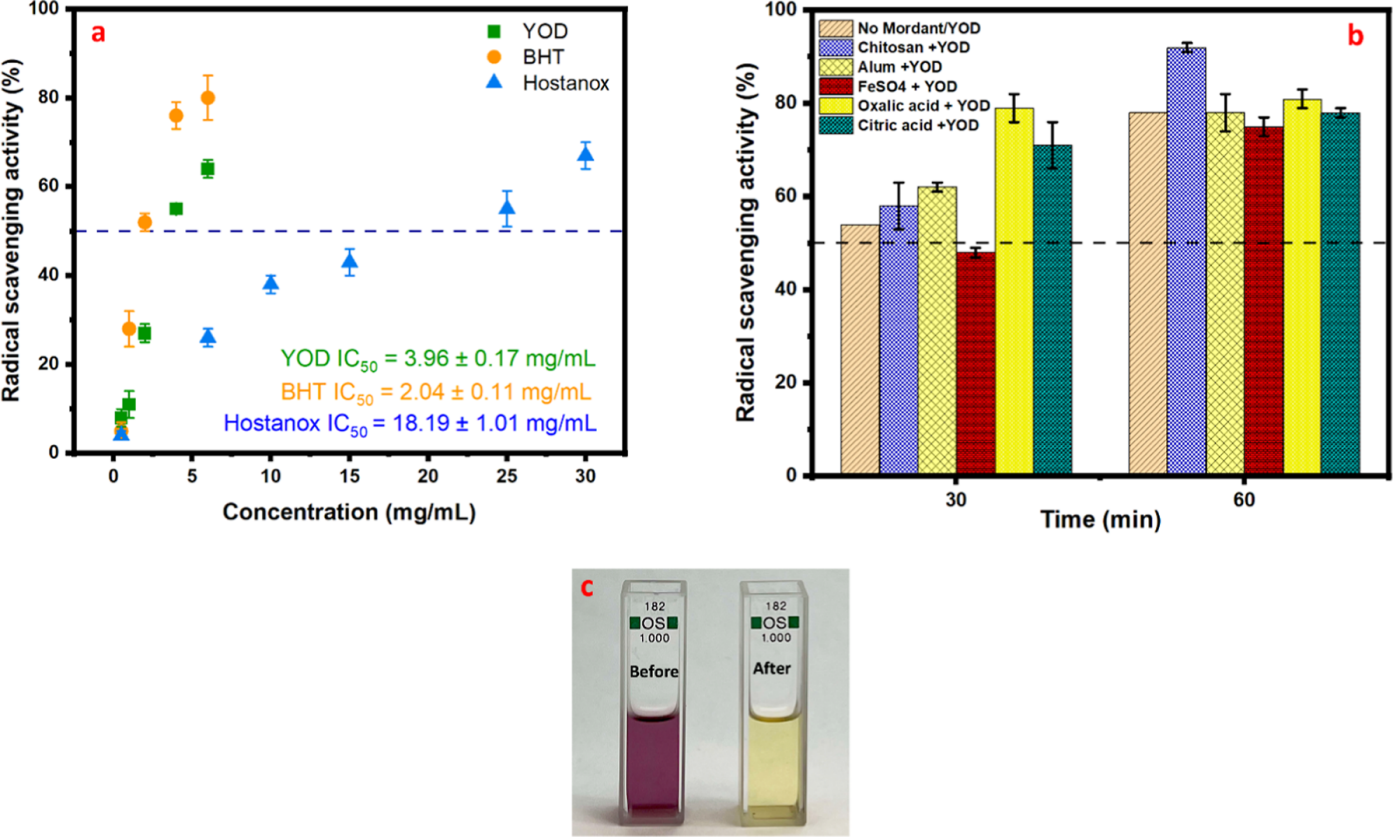
(a) Antioxidant activity of (a) YOD extract, BHT, and Hostanox with IC_50_ values, (b) radical scavenging activity in CNF films after 30 and 60 min, (c) appearance of DPPH solution before and after testing.

Since a lower IC_50_ value corresponds to higher antioxidant potency,^49^ BHT was the most effective in scavenging DPPH radicals, followed by YOD and then Hostanox. Although the YOD extract required a higher concentration to achieve the same level of radical scavenging as BHT, it still demonstrated significant antioxidant properties. This suggests that the YOD is a valuable natural antioxidant possessing considerable antioxidant properties.

To further assess the antioxidant ability of YOD extract, the radical scavenging activity (RSA) of CNF films dyed with YOD using different mordants was evaluated over 30 and 60 min, as shown in Figure 7b. The 2 wt % CNF film displayed no antioxidant activity. Therefore, it is not included in the results. However, all YOD-dyed films exhibited excellent antioxidant activity, with variations depending on the type of mordant used. Among them, CNF films mordanted with organic acids and chitosan demonstrated the highest RSA %, reaching approximately 80% and 90%, respectively, after 60 min of exposure to DPPH/methanol solution.

YOD extract is rich in quercetin and its glycosidic derivatives,^104^ which possess multiple hydroxyl groups capable of donating hydrogen atoms to neutralize free radicals, which leads to the interruption in oxidative chain reactions.^105^ In addition, Table S2 reveals that YOD extract contains phenolic acids (protocatechuic acid and trihydroxy-phenylglyoxylic acid), both of which are known for their effective antioxidant properties.^13^ Furthermore, the structural feature of flavonoids that are present in YOD, particularly the presence of galloylated derivatives present in the YOD (Table 1), contributed to enhanced radical scavenging activity due to the increased number of hydroxyl groups relative to glycosylated forms.^106^ Collectively, the abundance of hydroxyl-rich flavonoids, phenolic acids, and galloylated compounds explains the excellent antioxidant properties shown by the YOD extract.

The strong radical scavenging activity observed in YOD-dyed films mordanted with chitosan can be attributed to the combined synergistic effect of polyphenols present in YOD extract and the functional property of chitosan.^107,108^ The presence of chitosan enhances this effect by stabilizing and maintaining the antioxidant compounds on the CNF film, leading to improved overall antioxidant activity.^109^ Furthermore, the residual free amino (NH_2_) groups present in chitosan may contribute by interacting with free radicals to generate macromolecular radicals.^110,111^ The organic acids, oxalic acid and citric acid also showed excellent antioxidant activity. This has been reported mainly due to their metal chelating ability,^112,113^ where these acids can bind with metal ions known to catalyze oxidative reactions, which leads to the reduction of pro-oxidant metals and mitigates oxidative stress.^114^

The CNF films dyed without any mordant also exhibited strong antioxidant properties, scavenging almost 80% of the free radicals in 60 min. This suggests that the effective dyeing and functionalization of CNF films can be achieved without using any mordants, making the process more environmentally friendly. The high RSA % of these films indicates that YOD alone can provide both color as well as antioxidant protection, further supporting its potential application in active packaging and other functional materials where natural additives are preferred.

## Conclusions

This study highlighted that yellow onion skin extract is a promising natural dye for enhancing the functionality of cellulose-based materials. When applied to the CNF films, YOD showed excellent UV-shielding and antioxidant activities, supporting its potential use in biodegradable, active packaging materials. Among the natural mordants tested, chitosan significantly enhanced the dyeability and UV protection of CNF films dyed with the YOD, thereby offering an environmentally friendly alternative to metal mordants. The optimal condition for YOD adsorption onto CNF substrate was found to be at pH 5.8, as shown by the QCM-D study and zeta-potential measurements. UPF values further showed that chitosan-mordanted CNF films exhibited superior UV shielding capability compared to those of metal mordants. However, the cotton fabric did not achieve effective UV shielding with any dye-mordant combinations, due to the low dye amount in the fabric and the loose fabric structure.

For cotton fabrics, tannic acid as a natural mordant improved dye stability and color fastness properties. However, it did not increase the color strength compared to those obtained with metal mordants. Washing fastness test results at pH 10 and 7, with better performance under proposed pH 7 conditions, showed that new standards with milder, neutral washing conditions should be introduced to natural dyed textiles. This aligns with sustainability goals by reducing color loss and extending garment lifespan, and would be according to more environmentally benign laundry practices.

Preliminary in vitro skin sensitization tests revealed that Settonia extract was neither a skin sensitizer nor cytotoxic. However, the YOD extract demonstrated potential sensitizing effects at high concentrations. This emphasizes the necessity of assessing potential health risks alongside the functional benefits when developing natural dyes for commercial applications. Furthermore, it also highlights the need for additional studies with another sensitization assay to validate these results, as recommended by OECD guidelines.^115^

Yellow onion skins are a potential waste stream for further utilization as biobased chemicals for packaging and textiles. However, challenges in biomass collection and spoilage must be addressed. Therefore, future work should explore the pathways to valorization of the active compounds and evaluation of the benefits in terms of LCA and techno-economic feasibility. Current trends in unique and sustainable, well-designed textiles support small batch dyeing, in which yellow onion skins can offer a solution as a viable natural dye source. Our laboratory scale study showed the potential of the YOD and paved the way for further research and practical applications.

## Supplementary Material



## Data Availability

The data is available in the manuscript and the Supporting Information section. The Aalto University’s AI assistant tool was used to improve the language and readability of the manuscript. After using this tool, the authors reviewed and edited the content as needed and take full responsibility for the content of the publication.

## Notes

HPLC-MS TIC chromatogram of YOD extract (Figure S1); dyeing and mordanting conditions for CNF films (Table S1); HPLC-DAD-MS/MS data of *A. cepa* extract (Table S2); colorfastness result for cotton fabric dyed with Settonia as a dye using different mordants (Table S3); UPF for CNF films and cotton fabrics dyed with YOD and Settonia, respectively (Table S4); change in frequency and dissipation value (3rd overtone) obtained from QCM-D measurements for YOD and Settonia using different mordants (Figure S2) (PDF)

## #

VTT Technical Research Centre of Finland Ltd., PO Box 1000, Espoo FI-02044, Finland
