# Supplementary material for: Upcycling Agricultural Waste for Functional Interfaces: Yellow Onion Skin-Derived Dyes for Cellulosic Materials
Source: ACS Omega. 2025 Aug 11;10(32):36511–26. doi: 10.1021/acsomega.5c05183 (PMC12368664; doi:10.1021/acsomega.5c05183)
Supplement: Supplementary file 1 [file ao5c05183_si_001.pdf]

Supplemental information

Upcycling agricultural waste for functional interfaces: Yellow onion skin-derived dyes for cellulosic materials

Ritesh Sharma[1], Peppi Toukola[2], Juha Jordan[3], Julia Vuorinen[1,6], Ngoc Huynh[1], Nikita Durandin[4], Mikko Herrala[5], Anja Primetta[2], Jaana Rysä[5], Monika Österberg[1], Paula Nousiainen[1]*, Päivi Laaksonen[3], Riikka Räisänen[2]

[1] *Aalto University, School of Chemical Engineering, Department of Bioproducts and Biosystems, Vuorimiehentie 1, 02150 Espoo, Finland*

[2] *University of Helsinki, Craft Science, Siltavuorenpenger 5, 00014 Helsinki, Finland*

[3] *Häme University of Applied Sciences, HAMK Tech research unit, Vankanlähde 9, 13100 Hämeenlinna, Finland*

[4] *Tampere University, Faculty of Engineering and Natural Sciences, Korkeakoulunkatu 6, 33720 Tampere, Finland*

[5] *University of Eastern Finland, School of Pharmacy, Yliopistonrinne 3, 70211 Kuopio Finland*

[6] *Current address: VTT Technical Research Centre of Finland Ltd., PO Box 1000, Espoo FI-02044, Finland*

***Corresponding author:** Paula Nousiainen
Email address: paula.nousiainen@aalto.fi

List of tables and figures

Figure S1: HPLC-MS TIC chromatogram of the YOD extract detected at ESI negative mode. The peaks identified by their HRMS spectra at < 5 ppm accuracy are listed in Table 2. 2
Figure S2: Change in frequency and dissipation value (3rd overtone) (a) and (b) obtained from QCM-D measurements, on CNF thin films using YOD as a dye and chitosan as a mordant at pH 4 and 5.8. Change in frequency and dissipation value (3rd overtone) (c) (d), (e), and (f) obtained from QCM-D measurements, on CNF thin films using alum, $FeSO_4$, chitosan and tannic acid as a mordant at pH 4. 13

Table S1. Dyeing and mordanting conditions for CNF films with YOD..2
Table S2: HPLC-DAD-MS/MS data of the Allium cepa (cv. Settonia) extract. Peak numbers refer to the HPLC-UV-Vis chromatogram in Figure **1** in the manuscript. 3
Table S3. Dyeing results for cotton fabric with different mordants and Settonia as dye. 11
Table S4. UPF values and the UV protection category of CNF samples dyed with YOD solution and cotton fabrics dyed with Settonia...12

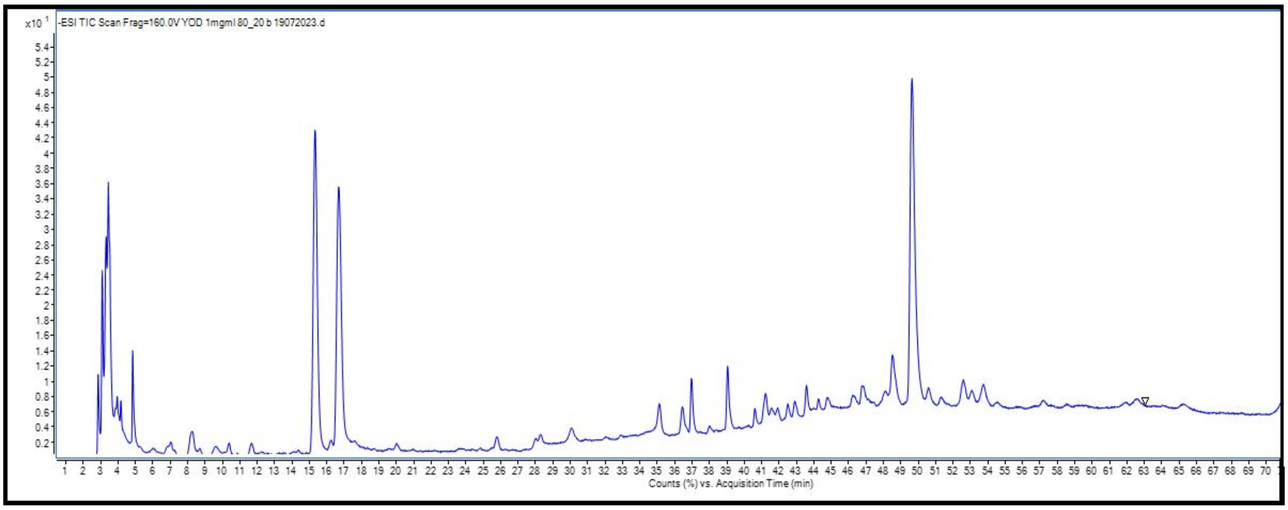

Figure S1: HPLC-MS TIC chromatogram of the YOD extract detected at ESI negative mode. The peaks identified by their HRMS spectra at < 5 ppm accuracy are listed in Table 2.

Table S1. Dyeing and mordanting conditions for CNF films with YOD.

Mordants	Dye concentration (g/L)	Mordant concentration (g/L)	LR	Mordanting/dyeing temperature (C)	Mordanting/ Dyeing time (min)
**Chitosan**	2	5	1:50	50/80	60/60
**Alum**	2	8	1:50	50/80	60/60
**$FeSO_4$**	2	3	1:50	50/80	60/60
**Oxalic acid**	2	5	1:50	50/80	60/60
**Citric acid**	2	8	1:50	50/80	60/60
**No mordant**	2	-	1:50	-/80	60/60

Table S2: HPLC-DAD-MS/MS data of the *Allium cepa* (cv. Settonia) extract. Peak numbers refer to the HPLC-UV-Vis chromatogram in Figure **1** in the manuscript.

**Peak No**	t_R **(min)**	λ_{max} **(nm)**	**[M + H]$^+$ (*m/z*)**	**[M + Na]$^+$**	**Other major ion**	**Fragment ions (*m/z*)**	**[M − H] (*m/z*)**	**[2M − H]$^-$ / [2M − 2H + Na]$^-$**	**Other major ion**	**Fragment ions (*m/z*)**	**Tentative identification**
**1**	6.9	228, 260, 294	155			[155]: 159 (2), 137 (18), 131 (2), 125 (4), 113 (4), 112 (7), 111 (100)	153			[153]: 109 (100)	Protocatechuic acid
**2**	9.4	230, 294	199			[199]: 229 (2), 218 (10), 171 (6), 153 (100), 151 (3), 139 (2)	197	– / 417		[417]: 397 (4), 219 (12), 201 (5), 197 (100), 153 (67), 151 (13)	Trihydroxy-phenylglyoxylic acid
						[199> 153]: 171 (100), 107 (38)				[417> 197]: 153 (100), 151 (7),125 (3), 109 (7)	
										[417> 153]: 107 (100)	
**3**	16.3	–	627			[627]: 465 (30), 303 (100), 257 (1), 247 (1) , 229 (2)	625			[625]: 463 (100), 343 (2), 301 (45), 300 (2)	Quercetin-di-*O*-hexoside
						[627> 465]: 303 (100), 285 (2), 274 (5), 201 (4), 149 (3), 143 (2)				[625> 463]: 301 (100), 179 (4)	
						[627> 303]: 201 (100)				[625> 301]: 255 (14), 229 (43), 205 (68), 193 (19), 179 (96), 151 (100), 121 (36)	
**4**	16.7	234, 294, 320sh	319	341	301	–	317	635 / –		[317]: 299 (36), 273 (4), 271 (5), 255 (18), 227 (3), 208 (5), 207 (21), 192 (3), 191 (100), 163 (22)	2-(3,4-Dihydroxybenzoyl)-2,4,6-trihydroxy-3(2H)-benzofuranone
										[317> 299]: 281 (3), 271 (23), 256 (2), 255 (100), 227 (22), 211 (11)	

Peak No	t_R (min)	λ_{max} (nm)	[M + H]$^+$ (*m/z*)	[M + Na]$^+$	Other major ion	Fragment ions (*m/z*)	[M − H] (*m/z*)	[2M − H]$^-$ / [2M − 2H + Na]$^-$	Other major ion	Fragment ions (*m/z*)	Tentative identification
										[317> 191]: 187 (42), 163 (100)	
**5**	17.3	–	627	649		[627]: 584 (5), 534 (5), 465 (55), 303 (100)	625			[625]: 463 (100), 343 (2), 301 (55), 300 (2), 261 (2), 179 (1)	Quercetin-di-*O*-hexoside
						[627> 465]: 345 (18), 312 (8), 303 (100)				[625> 463]: 301 (100), 300 (19), 151 (8)	
										[625> 301]: 273 (22), 179 (84), 151 (100), 107 (19)	
**6**	17.7	–	641	663		[641]: 621 (21), 479 (13), 409 (10), 325 (14), 317 (100)	639			[639]: 611 (5), 519 (14), 477 (23), 476 (11), 315 (100), 313 (40), 300 (6), 284 (9), 255 (8), 187 (6)	Isorhamnetin-di-*O*-hexoside
						[641> 317]: 228 (100)				[639> 314]: 300 (100), 298 (27), 271 (19), 242 (24), 169 (15), 151 (13)	
**7**	19.0	242, 290	599			[599]: 581 (52), 563 (100), 545 (52), 527 (45), 497 (34), 491 (29), 449 (53), 431 (85), 407 (30), 383 (29)	597			[597]: 507 (7), 477 (23), 459 (8), 441 (7), 433 (5), 417 (18), 387 (59), 369 (6), 357 (100), 315 (6)	Phloretin-di-*C*-hexoside
						[599> 563]: 527 (28), 509 (25), 491 (100), 476 (28), 449 (86), 431 (47), 413 (34)				[597> 387]: 369 (16), 327 (15), 315 (100), 245 (14), 239 (25), 209 (21), 167 (32), 153 (17)	
						[599> 527]: 473 (59), 461 (31), 431 (56), 407 (25), 395 (96), 377 (20), 367 (100), 361 (20)				[597> 357]: 289 (5), 251 (10), 209 (100), 191 (8), 167 (5), 165 (6), 137 (10), 123 (10), 122 (11)	
**8**	21.0	–	627			[627]: 607 (22), 590 (13) , 589 (53), 571 (18), 465 (27), 303 (100)	625			[625]: 605 (3), 561 (4), 454 (8), 454 (3), 453 (26), 317 (3), 315 (2), 301 (100), 243 (14), 179 (6)	Quercetin derivative

Peak No	t_R (min)	λ_{max} (nm)	[M + H]$^+$ (*m/z*)	[M + Na]$^+$	Other major ion	Fragment ions (*m/z*)	[M − H] (*m/z*)	[2M − H]$^-$ / [2M − 2H + Na]$^-$	Other major ion	Fragment ions (*m/z*)	Tentative identification
						[627> 589]: 571 (100), 561 (9), 543 (34), 473 (7), 409 (10), 311 (8), 273 (7), 271 (15), 245 (26), 217 (8)				[625> 301]: 179 (100), 151 (71)	
**9**	21.5	254, 265sh, 310sh, 366	465			[465]: 303 (100)	463	927 / –		[463]: 301 (100), 179 (1), 151 (1)	Quercetin-*O*-hexoside
						[465> 303]: 201 (27), 165 (95), 137 (100), 111 (50)				[436> 301]: 273 (16), 257 (4), 211 (6), 193 (5), 192 (4), 179 (75), 151 (100), 121 (3), 107 (5)	
**10**	22.7	–	–				615			[615]: 463 (95), 462 (13), 461 (100), 409 (6), 347 (5), 301 (77), 299 (33), 271 (8), 203 (5), 191 (4)	Quercetin galloyl hexoside
										[615> 301]: 298 (25), 270 (89), 257 (91), 254 (15), 238 (15), 229 (13), 199 (43), 179 (100), 151 (71)	
**11**	22.7	–	479			[479]: 317 (100), 302 (2)	477			[477]: 357 (2), 316 (5), 315 (100), 314 (23), 300 (9), 299 (6), 285 (2), 284 (3), 151 (3)	Isorhamnetin-*O*-hexoside
						[479> 317]: 341 (100), 302 (77), 285 (25)				[477> 314]: 300 (100), 299 (96), 285 (48), 284 (16), 272 (12), 271 (7), 243 (7), 169 (15), 151 (60), 107 (14)	
										[477> 300]: 272 (60), 271 (100), 256 (11), 255 (21), 243 (10), 228	

Peak No	t_R (min)	λ_{max} (nm)	[M + H]$^+$ (*m/z*)	[M + Na]$^+$	Other major ion	Fragment ions (*m/z*)	[M − H] (*m/z*)	[2M − H]$^-$ / [2M − 2H + Na]$^-$	Other major ion	Fragment ions (*m/z*)	Tentative identification
										(19), 227 (16), 161 (10), 151 (96), 150 (19)	
**12**	24.8	–	551			[551]: 531 (4), 526 (2), 465 (4), 303 (100), 257 (1), 229 (3), 213 (2), 165 (2)			505	[505]: 485 (1), 484 (2), 440 (1), 377 (2), 301 (100), 300 (5), 257 (2), 239 (1), 179 (1), 151 (2)	Quercetin malonyl hexoside
						[551> 303]: 284 (100)					
**13**	26.7	240, 250sh, 292	455			[455]: 437 (100), 303 (5), 302 (11), 301 (8), 273 (30), 165 (58)	453			[453]: 299 (100)	Protocatecoyl quercetin
						[455> 437]: 419 (11), 409 (14), 391 (4), 375 (7), 352 (6), 284 (6), 273 (25), 243 (4), 217 (7), 165 (100)				[453> 299]: 271 (100), 255 (6), 243 (6), 227 (4), 199 (4), 185 (6)	
**14**	27.6	240, 250sh, 292	455			[455]: 437 (100), 409 (2), 345 (4), 302 (15), 301 (17), 299 (2), 285 (5), 273 (40), 165 (69)	453			[453]: 301 (2), 299 (100), 271 (4)	Protocatecoyl quercetin
						[455> 437]: 448 (3), 409 (8), 407 (5), 285 (10), 273 (53), 256 (9), 165 (100), 155 (5)				[453> 299]: 271 (100), 255 (8), 243 (4), 215 (2), 211 (2), 199 (3), 145 (2)	
**15**	30.0	256, 298, 372	303			[303]: 273 (100), 257 (16), 246 (2), 245 (9), 228 (2), 165 (7), 153 (30), 137 (9), 111 (16)	301	603 / –		[301]: 299 (13), 273 (16), 272 (16), 271 (35), 257 (9), 255 (7), 179 (99), 151 (100), 121 (6), 107 (13)	Quercetin
						[303> 273]: 227 (100)				[301> 179]: 151 (100)	
**16**	31.3	–	765			[765]: 747 (36), 603 (100), 585 (60), 567 (6), 557 (5), 543 (5), 493 (6), 467 (12), 451 (5), 313 (7)	763			[763]: 611 (69), 601 (45), 599 (29), 557 (48),, 529 (26), 465 (71), 449 (100), 421 (38), 299 (49)	Quercetin dimer hexoside

Peak No	t_R (min)	λ_{max} (nm)	[M + H]$^+$ (*m/z*)	[M + Na]$^+$	Other major ion	Fragment ions (*m/z*)	[M − H] (*m/z*)	[2M − H]$^-$ / [2M − 2H + Na]$^-$	Other major ion	Fragment ions (*m/z*)	Tentative identification
						[765> 603]: 585 (100), 583 (18), 529 (47), 489 (19), 405 (31), 303 (43), 273 (19)				[763> 611]: 449 (50), 448 (100), 447 (47), 427 (35), 361 (45), 300 (28)	
**17**	35.0	256, 273, 304, 370	765	787		[765]: 613 (2), 603 (100), 601 (1), 585 (17), 543 (1), 467 (5), 451 (14), 423 (2), 341 (2), 303 (1)	763			[763]: 653 (1), 611 (100), 610 (1), 599 (4), 593 (3), 583 (3), 455 (1), 449 (27), 421 (4), 299 (2)	Quercetin dimer hexoside
						[765> 603]: 585 (37), 573 (15), 559 (9), 557 (20), 451 (100), 439 (9), 341 (25), 285 (9), 255 (9), 423 (35)				[763> 611]: 451 (7), 449 (100), 421 (10), 299 (4)	
						[765> 585]: 543 (100), 539 (25), 523 (6), 515 (42), 501 (13), 487 (24), 459 (16), 443 (10), 393 (20), 323 (11)				[763> 449]: 421 (100), 299 (7)	
**18**	35.3	256, 274, 303, 366	765	787		[765]: 603 (100), 585 (13), 559 (2), 493 (1), 467 (4), 451 (15), 433 (1), 423 (2), 341 (2), 303 (1)	763			[763]: 611 (100), 599 (1), 593 (1), 583 (2), 449 (42), 431 (5)	Quercetin dimer hexoside
						[765> 603]: 585 (60), 573 (3), 543 (9), 515 (21), 451 (100), 439 (7), 423 (44), 377 (22), 347 (23), 302 (4)				[763> 611]: 449 (100)	
						[765> 585]: 557 (22), 543 (100), 541 (96), 525 (10), 515 (11), 499 (13), 487 (20), 474 (28), 377 (24), 219 (79)				[763> 449]: 421 (100), 299 (4), 271 (7), 163 (7)	

Peak No	t_R (min)	λ_{max} (nm)	[M + H]$^+$ (*m/z*)	[M + Na]$^+$	Other major ion	Fragment ions (*m/z*)	[M − H] (*m/z*)	[2M − H]$^-$ / [2M − 2H + Na]$^-$	Other major ion	Fragment ions (*m/z*)	Tentative identification
**19**	38.0	–	287			[287]: 285 (17), 241 (23), 240 (100), 229 (18), 213 (30), 165 (28), 153 (44)	285			[285]: 283 (55), 258 (57), 257 (100), 239 (73), 229 (81), 213 (76), 187 (41), 185 (72), 163 (44), 155 (38), 151 (92), 141 (69), 123 (38)	Kaempferol
**20**	39.0	–	317			[317]: 302 (100), 301 (17), 298 (44), 286 (62), 285 (99), 284 (33), 283 (22), 269 (25), 163 (15)	315			[315]: 300 (100)	Isorhamnetin
						[317> 285]: 257 (100)				[315> 300]: 272 (25), 271 (38), 254 (17), 245 (14), 244 (12), 228 (13), 216 (23), 169 (13), 164 (29), 152 (18), 151 (100), 107 (17)	
**21**	40.8	250, 272, 304, 364	765			[765]: 603 (100), 585 (15), 567 (4), 557 (3), 557 (2), 539 (2), 405 (2), 313 (6), 303 (5), 273 (1)	763			[763]: 461 (9), 300 (3), 299 (100), 271 (21), 243 (7), 227 (4)	Quercetin dimer hexoside
						[765> 603]: 585 (83), 511 (12), 452 (15), 405 (11), 313 (100), 303 (10), 285 (17), 273 (11), 195 (17), 173 (19)				[763>299]: 271 (100), 243 (11), 227 (11)	
						[765> 585]: 511 (100), 503 (28), 433 (79)				[763> 271]: 244 (100)	
**22**	41.6	250, 289sh, 300, 366	603			[603]: 585 (40), 559 (10), 487 (5), 467 (14), 451 (100), 423 (31), 421 (4), 393 (5), 341 (9), 305 (9)	601			[601]: 553 (1), 547 (1), 490 (1), 475 (2), 449 (100), 436 (1), 431 (2), 421 (2), 358 (1), 299 (1)	Quercetin dimer or similar

Peak No	t_R (min)	λ_{max} (nm)	[M + H]$^+$ (*m/z*)	[M + Na]$^+$	Other major ion	Fragment ions (*m/z*)	[M − H] (*m/z*)	[2M − H]$^-$ / [2M − 2H + Na]$^-$	Other major ion	Fragment ions (*m/z*)	Tentative identification
						[603> 451]: 433 (8), 431 (3), 423 (100), 421 (9), 405 (7), 395 (3), 377 (4), 341 (10), 325 (3), 285 (7), 273 (3), 269 (2), 227 (3)				[601> 449]: 421 (100), 369 (2), 340 (2), 327 (2), 299 (4), 271 (4), 267 (2)	
**23**	43.1	–	765			[765]: 769 (3), 732 (2), 603 (100), 586 (3), 585 (5), 583 (6), 433 (3), 313 (2), 313 (2), 303 (2), 303 (17)	763			[763]: 724 (7), 609 (47), 461 (14), 449 (6), 299 (100), 271 (12), 270 (11), 243 (8)	Quercetin dimer hexoside
**24**	51.9	250, 272, 302, 364	603			[603]: 585 (100), 567 (19), 557 (24), 539 (14), 405 (16), 313 (74), 303 (55), 302 (9), 301 (9), 273 (24)	601			[601]: 583 (1), 461 (1), 299 (100), 271 (11), 255 (1), 227 (5)	Quercetin dimer
						[603> 585]: 567 (49), 557 (38), 550 (28), 539 (100), 521 (67), 493 (26), 455 (26), 433 (44), 405 (46), 323 (27)				[601> 299]: 271 (100), 255 (7)	
						[603> 313]: 269 (100), 265 (43), 257 (60), 241 (65), 229 (58)				[601> 271]: 216 (100)	
**25**	52.3	250, 272, 302, 364	603			[603]: 585 (75), 567 (22), 557 (25), 539 (9), 405 (13), 313 (68), 303 (100), 302 (13), 301 (12), 273 (14)	601			[601]: 299 (100), 271 (11)	Quercetin dimer
						[603> 303]: 285 (9), 274 (21), 273 (100), 257 (10), 245 (16), 200 (17)				[601> 299]: 271 (100), 243 (5)	
						[603> 585]: 567 (100), 557 (33), 539 (87), 521 (93), 493 (39), 459 (47), 433 (37), 397 (38), 313 (33)				[601> 271]: 216 (100)	

**Peak No**	t_R **(min)**	λ_{max} **(nm)**	**[M + H]$^+$ (*m/z*)**	**[M + Na]$^+$**	**Other major ion**	**Fragment ions (*m/z*)**	**[M − H] (*m/z*)**	**[2M − H]$^-$ / [2M − 2H + Na]$^-$**	**Other major ion**	**Fragment ions (*m/z*)**	**Tentative identification**
**26**	61.4	251, 275sh, 302, 360	903			[903]: 885 (27), 751 (90),, 603 (34), 597 (55), 585 (100), 573 (30), 303 (55)	901			[901]: 601 (59), 598 (62), 445 (100), 299 (45)	Quercetin trimer

Table S3. Dyeing results for cotton fabric with different mordants and Settonia as dye.

**Sample**	**CIELab**						**CC CIELab**		**Washing fastness**														**Rubbing fastness**	
**No.**	**L***	**a***	**b***	**K/S** Rmin	**K/S** 420 nm	**LF**	**ΔE** pH 7	**ΔE** pH 10	**CC** pH 7	**CC** pH 10	**WO** pH 7	**WO** pH 10	**PAN** pH 7	**PAN** pH 10	**PES** pH 7	**PES** pH 10	**PA** pH 7	**PA** pH 10	**CO** pH 7	**CO** pH 10	**CA** pH 7	**CA** pH 10	D(wf)	W(wf)
S1-0	74.94	3.76	17.99	4.80	1.33	5	5.59	7.62	3/4, Y	4, R	4	4/5	5	4/5	5	5	5	5	5	5	5	5	4/5	5
S2-Al	73.06	4.08	43.55	4.38	4.38	3	10.11	26.60	3/4	2	4/5	4	5	4/5	5	4/5	5	5	5	5	5	4/5	4/5	4/5
S3-Fe	47.39	1.98	16.03	6.87	5.59	4	3.85	15.46	4/5	2/3	4	4/5	5	5	5	5	5	5	5	5	5	5	4/5	5
S4-Tann	71.94	3.84	15.06	1.36	1.36	5	3.30	4.57	4, Y	4	3/4	4	4/5	5	5	5	4/5	5	5	5	5	5	4/5	4/5

LF = lightfastness, CC = colour change, WO = wool, PAN = polyacrylonitrile, PES = polyester, PA = polyamide, CO = cotton, CA = cellulose acetate, D(wf) = dry, along weft, W(wf) = wet, along weft

Table S4. UPF values and the UV protection category of CNF samples dyed with YOD solution and cotton fabrics dyed with Settonia.

Sample type	Mordant	UPF values	UV protection category
CNF film - No dye	No mordant - control	11.3	Insufficient
CNF Film-YOD solution	No mordant	62.9	Excellent
CNF Film-YOD solution	Chitosan	223.3	Excellent
CNF Film-YOD solution	Alum	31.2	Very Good
CNF Film-YOD solution	$FeSO_4$	28.7	Very Good
CNF Film-YOD solution	Oxalic acid	50.8	Excellent
CNF Film-YOD solution	Citric acid	33.2	Very Good
Cotton - Settonia	No mordant	7.2	Insufficient
Cotton - Settonia	Alum	6.5	Insufficient
Cotton - Settonia	$FeSO_4$	7.9	Insufficient
Cotton - Settonia	Tannic acid	7.6	Insufficient
Cotton - No dye	No mordant - control	4.6	Insufficient

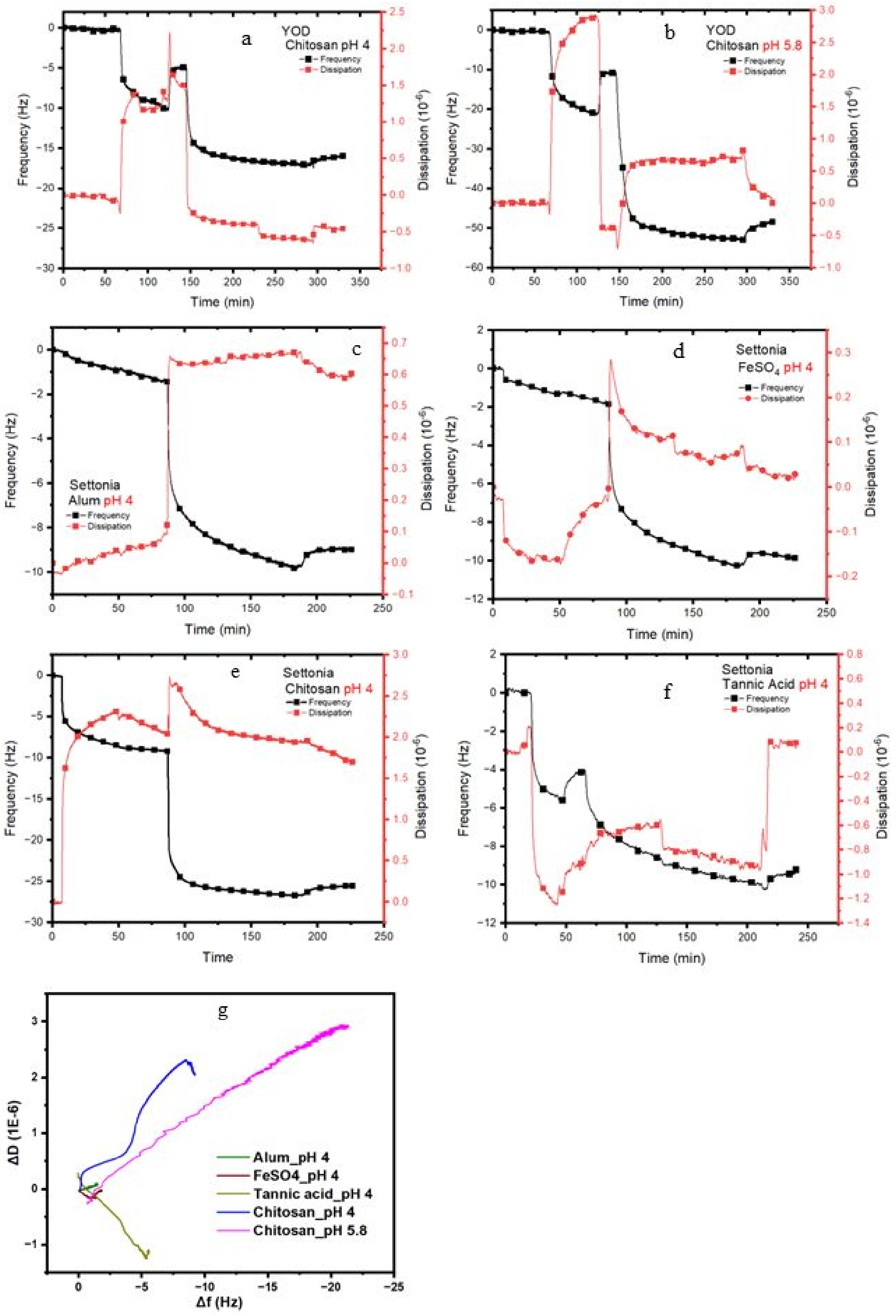

Figure S2: Change in frequency and dissipation value (3rd overtone) (a) and (b) obtained from QCM-D measurements, on CNF thin films using YOD as a dye and chitosan as a mordant at pH 4 and 5.8. Change in frequency and dissipation value (3rd overtone) (c) (d), (e), and (f) obtained from QCM-D measurements, on CNF thin films using alum, $FeSO_4$, chitosan and tannic acid as a mordant at pH 4, (g) Change in frequency vs dissipation value for all the mordants at pH 4 and 5.8.
